# The impact of environmental pollution on the quality of mother's milk

**DOI:** 10.1007/s11356-019-04141-1

**Published:** 2019-01-28

**Authors:** Martyna Pajewska-Szmyt, Elena Sinkiewicz-Darol, Renata Gadzała-Kopciuch

**Affiliations:** 1 Department of Environmental Chemistry and Bioanalytics, Faculty of Chemistry, Nicolaus Copernicus University in Toruń, 7 Gagarin St, 87-100 Toruń, Poland; 2 Interdisciplinary Centre for Modern Technologies, Nicolaus Copernicus University, 4 Wileńska St, PL-87100 Toruń, Poland; 3 Ludwik Rydygier Provincial Polyclinic Hospital in Toruń, Human Milk Bank, Św. Józefa 53-59, 87-100 Toruń, Poland; 4 Human Milk Bank Foundation, 128J Podkowy St, 04-937 Warsaw, Poland

**Keywords:** Breast milk, Polychlorinated biphenyls, Heavy metals, Cytokine

## Abstract

Breastfeeding is a gold standard of neonate nutrition because human milk contains a lot of essential compounds crucial for proper development of a child. However, milk is also a biofluid which can contain environmental pollution, which can have effects on immune system and consequently on the various body organs. Polychlorinated biphenyls are organic pollutants which have been detected in human milk. They have lipophilic properties, so they can penetrate to fatty milk and ultimately to neonate digestive track. Another problem of interest is the presence in milk of heavy metals—arsenic, lead, cadmium, and mercury—as these compounds can lead to disorders in production of cytokines, which are important immunomodulators. The toxicants cause stimulation or suppression of this compounds. This can lead to health problems in children as allergy, disorders in the endocrine system, end even neurodevelopment delay and disorder. Consequently, correlations between pollutants and bioactive components in milk should be investigated. This article provides an overview of environmental pollutants found in human milk as well as of the consequences of cytokine disorder correlated with presence of heavy metals.

**Figure:**
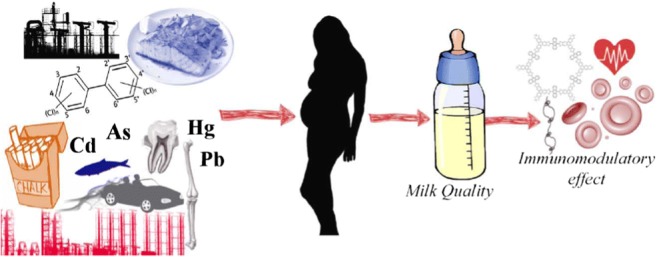
Graphical abstract

Graphical abstract

## Introduction

**Fig. 1:**
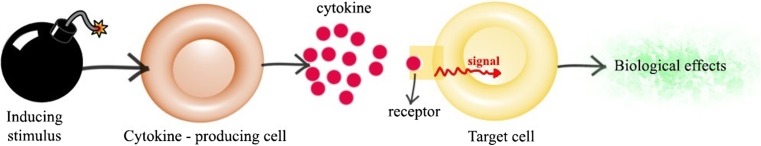
Mechanism of cytokine production and action

A newborn child is exposed to many factors which may have negative impact on its health. Thus, protection is very important during infancy. One of its elements is breastfeeding, which reduces frequency of diarrhea and the risk of such diseases as necrotizing enterocolitis (NEC). Benefits of breastfeeding can be seen both during infancy and later in adult life (Duijts et al. 2010; Le Huёrou-Luron et al. 2010; Martin et al. 2005; Owen et al. 2003). Ingredients contained in milk have many functions, such as providing nutrients and energy; thanks to the presence of specific proteins, or oligosaccharides milk also has bioactive properties as these compounds have an impact on the development of the child’s immunity (Gomez-Gallego et al. 2016). Among the compounds associated with the immune system are cytokines, which are polypeptides that operate in a complex network. Connection to a specific receptor produces an immunomodulating effect (Fig. 1) (Garofalo 2010).

In newborn babies, the full-scale production of cytokines begins with a certain delay, and this is why their presence in women’s milk is so important. Delivered with mother’s milk, they interact with respiratory and digestive tract cells and act as anti-inflammatories and immunomodulators (Meki et al. 2003).

Unfortunately, the effect of negative factors such as stress or toxic compounds (i.e., heavy metals) can cause disruption in the production of cytokines (Kendall-Tackett 2007; Krocova et al. 2000). The recent reports suggest that high concentrations of pro-inflammatory cytokines in milk may be connected to inflammation in the child, and their excess in food can be harmful when the newborn has necrotizing enterocolitis (MohanKumar et al. 2017; Rentea et al. 2017). Monitoring mother’s milk is very important both in the search for compounds crucial for a developing organism and in testing for potential contaminants—environmental agents which can disrupt developmental process (Table 1) such as heavy metals, polychlorinated biphenyls, or dioxins (Rebelo and Caldas 2016; Urbaniak et al. 2015 ). Persistent organic pollutants such as dioxins and polychlorinated biphenyls are very hard to eliminate from the environment. They are lipophilic, i.e., they accumulate in adipose tissue. These compounds could be transferred to infants through breast feeding (Man et al. 2017). For newborns, these substances are particularly dangerous due to the immaturity of internal organs and the nervous system. Maternal exposure to heavy metals as Pb or Hg and persistent organic pollutants were associated with children neurodevelopment delay (Čechová et al. 2017; Kim et al. 2018; Shelton et al. 2014). Environmental pollutants induce changes in structure of immune system and also in function by disturbing the homeostasis. The toxicants cause stimulation or suppression of the immunomodulatory components and can influence indirectly on the various body organs and other system as nervous, reproductive, respiratory, and endocrine (Bahadar et al. 2015; Mokarizadeh et al. 2015).

**Table 1:** Anthropogenic pollutants and examples of toxic effects

Class of compounds	Examples of toxic effects	Literature
Pesticides • Organochlorine pesticides (OCPs) • Organophosphate pesticides (OPPs)	Delayed effects on central nervous system functioning	Ennaceur et al. 2008
	Disruption of endocrine system (hormonal imbalance)	Eskenazi et al. 2006
	Increased risk of cancer	Ribas-Fitό et al. 2006
	Genotoxic effects	Yamazaki et al. 2018
	Abnormal behavior	
	Growth retardation	
Organochlorines • Polychlorinated dibenzo-dioxin (PCDDs) • Polychlorinated dibenzofurnas (PCDFs) • *Polychlorinated biphenyls (PCBs)	Dermatitis	Gascon et al. 2013
	Disorders in the endocrine and reproductive system	Hansen et al. 2014
	Neurological and behavior problems	Passatore et al. 2014
	Metabolic diseases (diabetes, obesity)	Tang-Péronard et al. 2014
	Reduced immune response	Taylor et al. 2013
	Increased risk of asthma	
Bisphenols	Neuroendocrine disorders (e.g., precocious puberty)	Braun et al. 2011
	Obsesity	Rochester 2013
	Diabetes	
	Anxiety	
	Hyperactivity	
Parabens	Endocrine related disorders: (e.g., obesity, thyroid gland disorders, female/male reproduction issues)	Nowak et al. 2018
Phtalates	Adverse neurodevelopmental effects (e.g., autism spectrum disorders)	Benjamin et al. 2017
	Reproductive toxicity (testicular cancer, male infertility, reproductive abnormalities)	Katsikantami et al. 2016
	Asthma and allergic symptoms, overweight, and obesity	
Brominated flame retardants	Endocrine disruption	Műller et al. 2016
	Neurodevelopment and behavioral disorders	
	Potentially increased risk of cancer	
Perofluoroalkyl substances • Perfluorooctane sulfonate (PFOS) • Perfluorooctanoate (PFOA)	Delayed effects on development	Granum et al. 2013
	Decreased antibody response	
*Heavy metals: • Cadmium (Cd) • Arsenic (As) • Lead (Pb) • Mercury (Hg)	Immunotoxicity (weakening of the immune system)	Grandjean and Landrigan 2006, 2014
	Toxic effect on neurodevelopment	Samiee et al. 2019
	Development of autoimmune diseases (i.e., allergies or atropy)	
	Clinical disorders (e.g., anemia, cancer, reproductive disorders, depression	

*One of the main topics described on this paper

Human milk monitoring makes it possible to assess the exposure of the mother and the baby. This is a non-invasive way to track environmental pollution (Lopes et al. 2016; Rebelo and Caldas 2016) and it is recommended by WHO.

The aim of this study is to summarize the current knowledge regarding monitoring human milk for the presence of compounds that could pose a threat to the health of both mothers and children, and linking their presence in milk to immunomodulatory compounds. It is important to summarize the latest achievements and current knowledge on pro-inflammatory cytokines in the context of biomarkers of inflammatory conditions in breastfeeding women and their double role: ingredients essential for a vulnerable child (immunomodulatory function) and compounds that may harm infant’s digestive tract in case of necrotizing enterocolitis.

Because each of the review points could be a separate and extensive paper, the purpose of our work was to highlight key informations about discussed problems, and show that environmental pollutants can be associated with cytokine profile in breast milk, which can have harmful effect on newborn child.

A systematic review was conducted using PubMed and Scopus databases. Search strategies include keywords as “polychlorinated biphenyls,” “PCBs,” “human milk,” “breast milk,” “cytokine,” “heavy metals,” “lead,” “mercury,” “cadmium,” and “arsenic” in various combination. We limited our paper to articles published in the English language. We performed the last search on 2 December 2018.

## Human milk composition

**Fig. 2:**
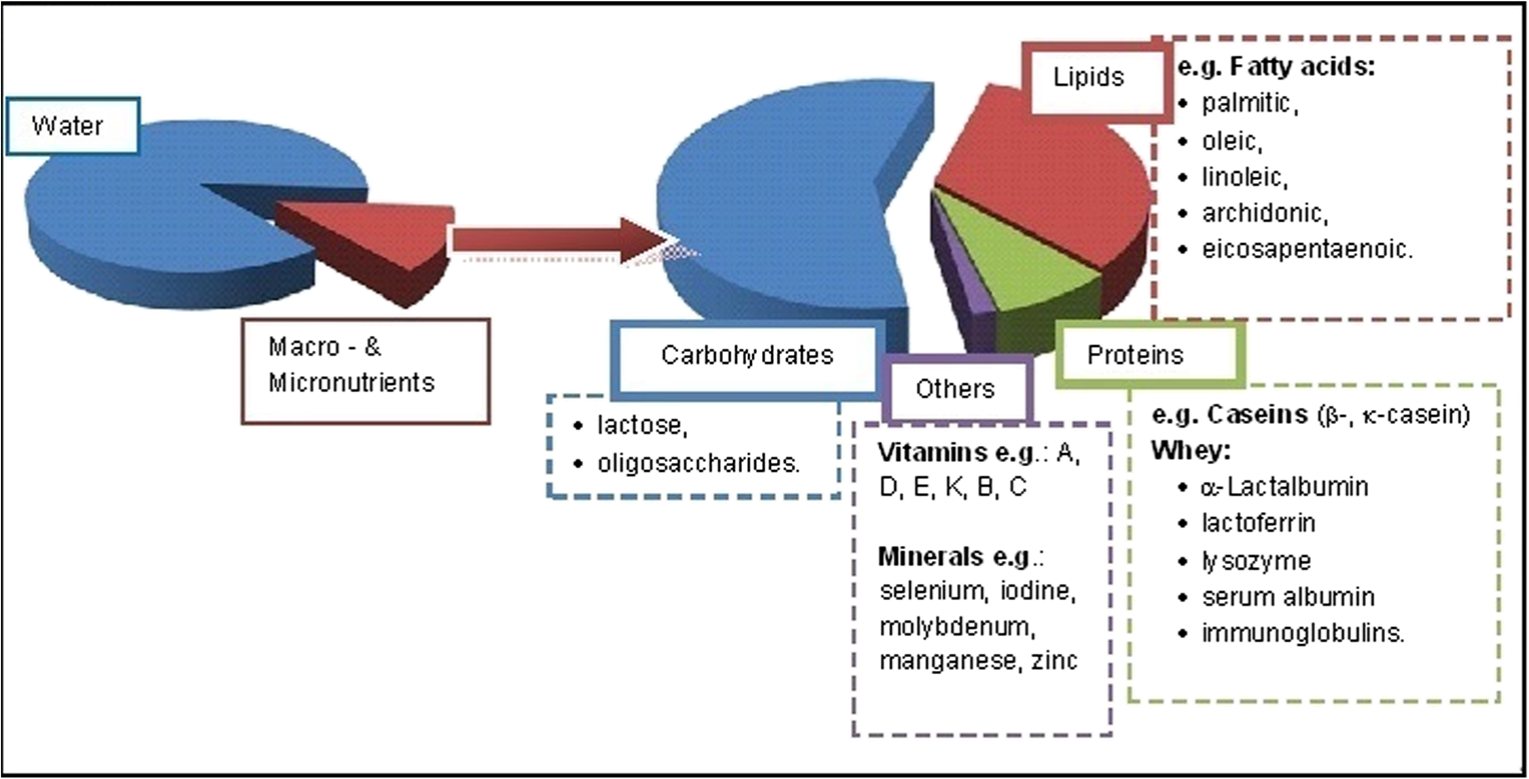
Composition of human milk

Human milk is referred to as the golden standard of nutrition. It has unique composition: it is in 87% made up of water, and the rest is macro- and micronutrients—7% carbohydrates (mainly lactose), 4% lipids, and 1% proteins and others (vitamins and minerals) (Fig. 2). Milk composition changes with lactation periods, which is caused by physiological factors and also corresponds to the current needs of the infant. At the beginning of the lactation period, colostrum is produced (for 3–4 days), then immature milk (for about 2 weeks), and finally mature milk. Colostrum is rich in protein and vitamins such as A, B_12_, and K as well as oligosaccharides. Concentration of these compounds in colostrum is higher than in mature milk. Colostrum is intended to provide immune protection to a child against numerous environmental pathogens. In mature milk, concentration of lipids is higher than in colostrum. Milk ingredients not only ensure proper growth and development of the baby, but also due to their bioactive properties they contribute to and support defense mechanisms, and thus they are essential to the health of the infant. Additionally, milk has antioxidant properties. However, the composition of human milk is affected also by such external factors as mother’s diet, lifestyle, and potential environmental pollutants (Andreas et al. 2015; Emmett and Rogers 1997; Gomez-Gallego et al. 2016; Mandal et al. 2014; Matos et al. 2015).

## Organic pollutants in milk

**Fig. 3:**
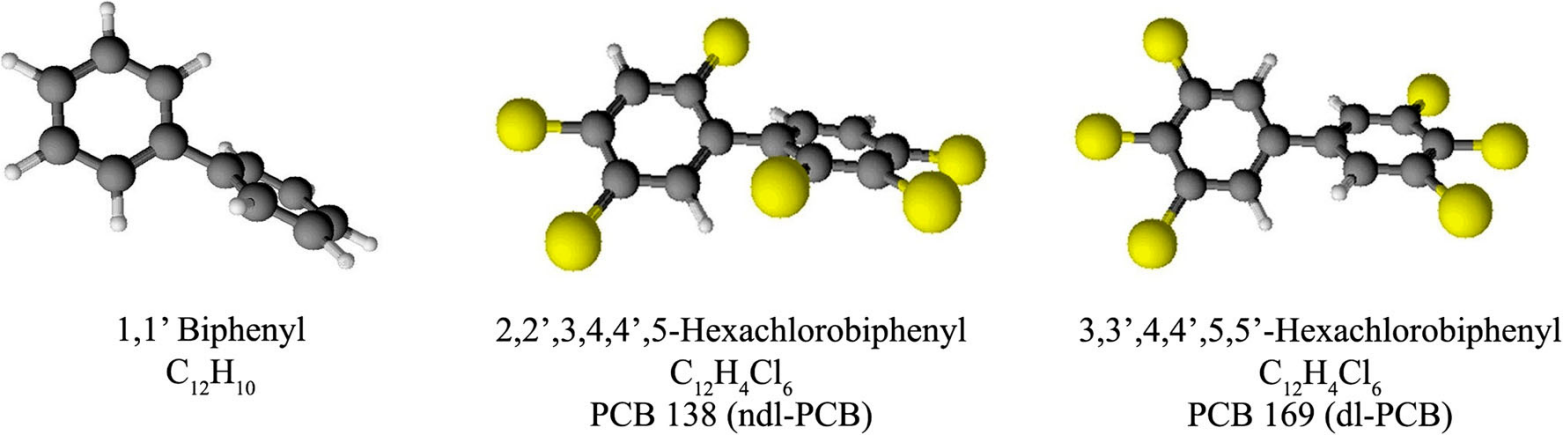
Structure of polychlorinated biphenyls

Because of high fat content in milk, currently it may be difficult to eliminate from it such lipophilic compounds as dioxins: polychlorinated dibenzo-dioxin (PCDDs), polychlorinated dibenzofurans (PCDFs), and polychlorinated biphenyls (PCBs) (Lopes et al. 2016). The last mentioned group—PCBs—is one of the main topics discussed in this paper. Polychlorinated biphenyls are a group of compounds comprising 209 congeners. They are built of two phenyl rings combined by a C–C bonding (Andersson et al. 1997a). Individual congeners differ by the number of attached chlorine atoms. The arrangement of the rings depends on the number and position of chlorine atoms (Fig. 3). The flat conformation has biphenyl without chlorine in ortho position (non-ortho). Mono-ortho and di-ortho PCBs have similar structures. This group of congeners is called dioxin-like PCBs (dl-PCBs 77, 81, 126, 169, 105, 118, 156, 157, 123, 167, 189). The chlorination substitution of PCB rings influences the toxicity of compound. Their structure and toxicity is similar to a very dangerous congener 2,3,7,8-Tetrachlorodibenzo-p-dioxin (2,3,7,8—TCDD), which is the most toxic compound in dioxin class. The toxic and biological effects of these environmental agents (teratogenic and carcinogenic effects) are connected with their tendency to bind with aryl hydrocarbon receptor (AhR), the cellular protein (ligand-activated transcription factor); thus, TCDD currently occupies the first place in the toxic equivalency factor (TEF) table. The Health and Environmental organizations advise to use this factor method for estimating health hazard connected with exposure to these compounds (Parvez et al. 2013). The TEF value is determined for particular compounds as PCBs, which have toxicity and biological effects relative to TCDD (TEF = 1), which is reference substance. It is also possible to assess toxic effect of mixture of dioxins. The total toxic equivalent (TEQ) is calculated by concentration of an individual compounds with TEF, and resulting are summed up as WHO-TEQ (Van den Berg et al. 2006). The non-dioxin-like PCBs (ndl-PCBs: 28, 52, 101, 138, 153, 180) show different toxic properties; however, they are very stable and are the substances most likely to accumulate (Andersson et al. 1997a, 1997b; Faroon et al. 2003; Van den Berg et al. 2013). This group of PCBs is called as indicator PCBs and they occur predominantly in the environment. Polychlorinated biphenyls are very hard to eliminate from the environment; for example, half-lives of the indicator PCBs are PCB 28, 5.5 years; PCB 52, 2.6 years; PCB 101, 2.8 years; PCB 118, 11.5 years; PCB 138, 12 years; PCB 153, 17 years, PCB 180, 15 years (Bányiová et al. 2017; Bu et al. 2015; Ritter et al. 2011).

**Fig. 4:**
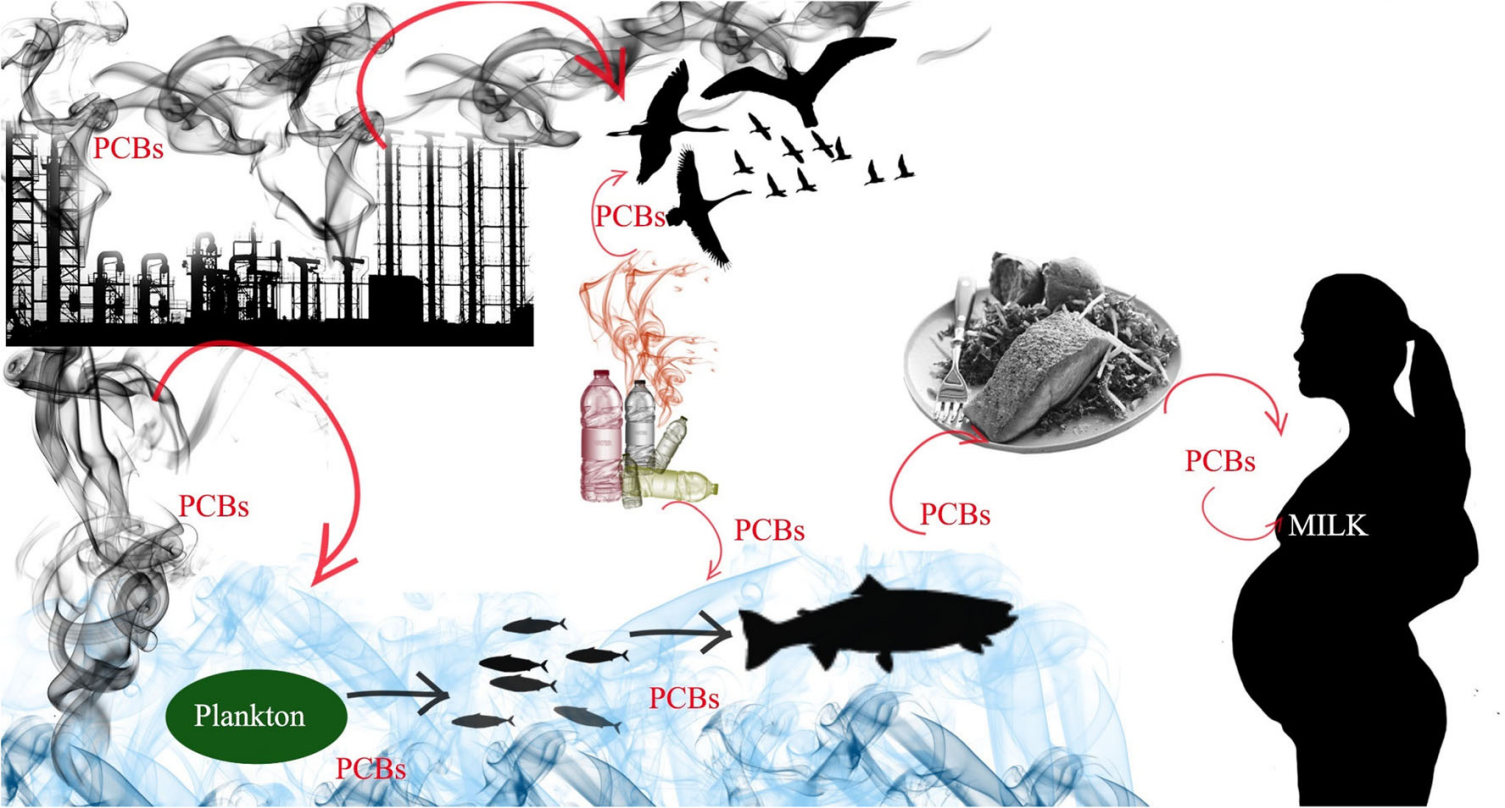
Sources of exposition and bioaccumulation of PCBs

The consequences of widespread pollution with these compounds and the threat PCBs pose to living organisms are a result of the multitude of ways in which this compound was used for over 50 years. Polychlorinated biphenyls found applications, e.g., in electrical insulation, lubricating and hydraulic fluids, as plasticizers for plastic and paints, and as additives in glue and copy paper (Erickson and Kaley 2011). Commercial production of these compounds was banned in the USA in the late 1970s. Unfortunately, due to PCBs’ resistance to decomposition processes, even today, most of the lakes and rivers are polluted (Paliwoda et al. 2016; Rocheleau et al. 2011). Furthermore, illegal burning of hazardous waste, for example, old transformers containing chlorinated hydrocarbons, increases this type of pollution in the environment (Asamoah et al. 2018; Rivezzi et al. 2013). Contamination is carried by atmospheric transport over long distances to the regions which are not in the immediate vicinity of industrial plants (Gevao et al. 1998; Nelson et al. 1998; Norstrӧm et al. 2010) . An important problem is penetration of these compounds into water: due to their lipophilic properties, they bioaccumulate in fish. The species at the top of the food chain are the most vulnerable (Fig. 4). The fish are an essential source of omega 3 fatty acids, which are not synthesized by mammals but are necessary for proper metabolism. Consequently, the consumption of fish and fish products by humans and domesticated animals ensures the delivery of docosahexaenoic acid (DHA), yet it also creates the risk of exposure to harmful organic compounds that may be present in such food (Paliwoda et al. 2016).

Research conducted in Spain found that fish products are the main source of PCB, dioxin, and furan consumption, while the contribution from grains and vegetables is small (Marin et al. 2011). Fish consumption can cause accumulation of organic pollutants in tissue, and breastfeeding is the main way of excreting such substances from a woman’s body (Uemura et al. 2008). The most polluted species are sardine, red mullet, and mackerel (Perellό et al. 2015), and fish with a lot of fatty tissue (salmon and trout) (Struciński et al. 2013). It is safer to eat fish from lower trophic levels. The big fish are exposed to higher PCB concentrations due to bioaccumulation and biomagnification (Paliwoda et al. 2016). People who have diet low in fish can fill the lack in DHA deficiency with fish oil supplements. Such supplements usually come in the form of microcapsules in which unstable fatty acids are protected against degradation (Barrow et al. 2009). However, there remains a question regarding pollutants in these capsules: Is oil derived from fish and enriched with omega-3 fatty acids also a source of lipophilic impurities? Unfortunately, if the oils have not been cleaned enough, the contents of PCBs or dioxins may exceed the limits, particularly if the oils come from fish that are on the highest trophic level, for example, shark. Another problem are differences in concentrations of these compounds in the product of the same type, but coming from a different manufacturing process (another region or collection season) (Fernandez et al. 2006; Martί et al. 2010; Rawn et al. 2009).

Mother’s milk is a non-invasive matrix, which contains information about exposition to organic pollution. For instance, one study (Chen et al. 2015) showed that concentration of impurities in milk is correlated with levels of impurities in the feces of a neonate who has been fed this milk.

In order to assess the exposure of milk to these compounds, a number of factors should be considered that may influence their deposition in the body. These factors include mother’s age, BMI before pregnancy, weight gain during pregnancy, her weight at birth (Lignell et al. 2013), smoking habit, area of residence (a neighborhood with industrial plants has the biggest impact) (Černá et al. 2010; Schuhmacher et al. 2007), and a detailed diet description, e.g., in the case of fish consumption, it is important how frequently the fish are eaten and what they are (fat or lean, fresh or frozen, local or imported) (Skrbić et al. 2010). Quantity and time of sampling from one mother are also very important factors, because organic pollutants such as PCBs and PCDD/Fs can be present in milk until the end of lactation (Vigh et al. 2013). Therefore, the best way is to conduct analysis during at least two or three stages of lactation (Uemura et al. 2008; Wang et al. 2008). Analysis of environmental pollutions in women’s milk has been conducted by many researchers from the whole world, and in the most cases they focused on specific areas of the following states: Sweden (Lignell et al. 2013), Spain (Schuhmacher et al. 2007), the Czech Republic (Bencko et al. 2004; Černá et al. 2010), Hungary (Vigh et al. 2013), Slovakia (Čechová et al. 2017; Chovancová et al. 2011), Russia (Mamontova et al. 2017; Polder et al. 2008), Ireland (Pratt et al. 2012), Poland (Szywrińska and Lulek 2007; Škrbić et al. 2010), Greece (Costopoulou et al. 2006), Tunisia (Hassine et al. 2012), Canada (Ryan and Rawn 2014), and China (Deng et al. 2012; Zhang et al. 2016; Zhang et al. 2017), Ghana (Asamoah et al. 2018), Netherlands, Norway (Čechová et al. 2017), The Republic of Moldova (Tirisina et al. 2017), France, Denmark, Finland (Antignac et al. 2016), Croatia (Klinčić et al. 2016). Generally, the number of published papers between 1979 and 2017 (searching in Scopus database; using keywords as “polychlorinated biphenyls,” “breast milk,” “human milk,” and “monitoring”) are 169, which include documents by country USA (38), Japan (20), Germany (19), Sweden (15), and China (13) (top five) (Scopus database, 8.04.2018). The amount of research on milk is justified as milk is the first natural food for a baby, which is a very important source of essential compounds; however, when the mother is exposed to organic pollutants, her milk can be also a source of this impurities. The tolerated daily intake of PCDD/PCDF/PCB (according to WHO) should not exceed 1–4 pg/kg bw/day, equivalent milk level 0.2–0.9 pg/g lipids (Van den Berg et al. 2017).

PCBs conformation influences on the tendency to bind with aryl hydrocarbon receptor (AhR), where the highest properties have a dioxin-like polychlorinated biphenyls. Non-dioxin-like polychlorinated biphenyls have effects on the immune system, for example, can cause production of reactive oxygen species (ROS) in human neutrophil granulocytes, which was investigated by Bernsten et al. (2016). Their research shows that three PCBs (52, 153, and 180) induced the production of ROS. Long-term exposure to organic pollutants can lead to serious health consequences such as dermatitis, disorders in the endocrine (e.g., impact on the thyroid function), and reproductive system, and neurological problems (Passatore et al. 2014). Maervoet and co-workers (Maervoet et al. 2007) presented the results of research on relationships between organochlorine pollutions and thyroid hormone levels in cord blood (*n* = 198 neonates). The conclusion of this paper is that impurities such as PCBs may affect triiodothyronine (fT3) and free thyroxine (fT4) hormones and consequently the thyroid system of infants. This is why care is necessary during children’s development, especially in the case of vulnerable newborns, whose immunological system is still forming. Weakness of the immune system can lead to allergy, asthma, and infection (Gascon et al. 2013; Lignell et al. 2013). The PCB compounds have been also linked with behavioral problems in children; i.e., prenatal exposure to PCB 153 is associated with anxiety and attention deficits among children, which was researched by Verner and co-workers (Verner et al. 2015) in epidemiological studies. In the Norwegian Mother and Child Cohort Study (including 1024 children) conducted by Caspersen et al. 2016a and Caspersen et al. 2016b, the results show that low-level maternal exposure (PCB 153 was 0.8 ng/kg bw/day; range 0.1–17) to PCB with six chlorine atoms such as 153 is associated with girls’ poorer expressive language skills in early life. Language development delay in girls and problems with using complete grammar structures (44,092 children included in the study) were correlated with intake of PCB 153 (median 11 ng/kg bw/day to 5–28 ng/kg). The results of milk analyses from different countries are given in Table 2, which shows the number of samples and comments. In most cases, the dominant forms of accumulated PCBs are biphenyls with six or more chlorine atoms No. 138, 153, 180 (Černá et al. 2010; Chovancová et al. 2011; Hassine et al. 2012; Lignell et al. 2013; Schuhmacher et al. 2007). Unfortunately, such number of chlorine atoms causes resistance to being metabolized and results in greater accumulation (Faroon et al. 2003; Skrbić et al. 2010). Comparison of these data is very difficult, because in every case the sample have different parameters as period of lactation, years, volume, and sample preparation method. Furthermore, in Table 2, we include result obtained for total analyzed PCB. In one, researchers investigated only indicator PCBs, in other more than seven PCBs. The aim was to show on what scales these tests can be conducted. In our opinion, study performed for indicator PCBs are enough, because these congeners have been used as indicators of the total PCBs content. The non-dioxin-like PCBs are used on the basis environmental analysis. Indicator PCBs were selected as representatives for all PCBs, they occur predominantly in biotic and abiotic matrices (Baars et al. 2004).

**Table 2:** Monitoring of organic pollutants—PCBs in human milk

Number of tested PCBs	Country	Number of mothers	Years	Concentrations (ng/g lipid)	Comments	Reference
Di-ortho PCBs	Sweden	413	1996–2010	∑_3_ 15–363	The higher concentration of PCBs, the higher weight of the baby after birth.	(Lignell et al. 2013)
35 PCBs	Czech Republic	90	1999–2000	∑_35_ 293–13.754	High concentrations found in milk from mothers who live in industrial areas, PCB concentrations increased with mother’s age.	(Černá et al. 2010)
33 PCBs	Spain	20	2012	∑_33_ 121–471	Concentration of POPs was higher in milk from women living in urban areas than in industrial areas. The author suggests this may be caused by urban women eating a larger number of fish dishes.	(Schuhmacher et al. 2007)
19 PCBs	Hungary	22 (3× at 5, 12, 84 lactation days)	2012	∑_19_ Mean 5 days, 37.45; 12 days, 30.66; 84 days, 30.08	PCB concentration decreased during lactation. The biggest fall occurred between day 5 and 12.	(Vigh et al. 2013)
12 PCBs	Slovakia	33	2006–2007	∑_12_ 2.7–32.0 TEQ pg/g	In milk from women who live in industrial areas, concentration of POPs exceeded TDI limit (WHO).	(Chovancová et al. 2011)
8 PCBs	Tunisia	36	2010	∑_8_ 16.4–1360.2	PCB concentration was positively correlated with the age of mothers, who gave birth for the first time Mothers with second-born and following children - no correlation.	(Hassine et al. 2012)
18 PCBs	China (Shenzhen)	60	2007	∑_12 dl-PCBs_ 1.964–13.967 ∑_6 indicate PCBs_ 0.0034–0.0392	Body burden of PCBs was positively correlated with the period of residence in Shenzhen and fish consumption. Positive correlation between mother’s age and body burden for DL-PCBs and PCBs.	(Deng et al. 2012)
7 PCBs	Ghana	128	2014–2016	∑_7indicate PCBs_ 3.64	In an electronic waste hot spot area, mean concentration of PCB was much higher than in non-spot area (4.43/0.03)	Asamoah et al. 2018

The dominant method used for detection and identification of polychlorinated biphenyls and dioxins is gas chromatography with electron capture detector (Hassine et al. 2012; Polder et al. 2008; Skrbić et al. 2010), and mass spectrometry (Bencko et al. 2004; Černá et al. 2010; Chovancová et al. 2011; Deng et al. 2012; Ryan and Rawn 2014; Schuhmacher et al. 2007; Vigh et al. 2013; Zhang et al. 2016). Techniques used for sample preparation include classical liquid-liquid extraction (LLE) (Bencko et al. 2004; Černá et al. 2010; Chovancová et al. 2011; Hassine et al. 2012; Ryan and Rawn 2014), solid phase extraction (SPE) (Dmitrovic and Chan 2002; Lin et al. 2016), and accelerated solvent extraction (ASE) (Deng et al. 2012; Vigh et al. 2013; Zhang et al. 2016). It is important to improve the sample preparation stage and search for a method which will make it possible to analyze a lot of samples in a short time, with small amounts of solvents. An interesting approach was a QuEChERS technique (Luzardo et al. 2013; Asamoah et al. 2018) where the extract was cleaned by dispersive solid phase extraction (dSPE).

## Cytokines in milk

It is well known that breastfeeding provides bioactive ingredients for proper development of a child. The unique composition of human milk was highlighted in many papers (Andreas et al. 2015; Bode 2012; Gao et al. 2012; Lӧnnerdal 2016; Walker 2010). These bioactive ingredients include cytokines, which in newborns are not produced in sufficient amounts. Because milk cytokines work not only through the digestive tract but also get into the bloodstream of the newborn, they compensate for this deficit.

Cytokines are a group of protein, which shows important functions in regulating inflammation (Quaqliato and Nardi 2017). These compounds have pleiotropic properties and work in complicated network. Depending on the target cell, a single cytokine may cause or inhibit signal (Arai et al. 1990). These small proteins are present in the most biological processes as disease pathogenesis, specific response to antigen or non-specific response to infection, embyronic development and stem cell differentiation, and other major pathways (Dinarello 2007). These signaling molecules include chemokines, interleukins interferons, and growth factors (Agarwal et al. 2011). Their presence in human milk provides anti-inflammatory protection and immunomodulating effect—activation and retention of the immune response at the right time (Amsen et al. 2009; Bryan et al. 2016; Meki et al. 2003). Unfortunately, it is risk that cytokines after infection does not return to their normal state and still are present in body fluids with high concentrations. Consequently, disregulation of cytokine production can have harmful effects on organism function (Dinarello 2007). Therefore, we should ask a question: What are the effects of the imbalance of cytokine production and their high concentration in milk?

When studying the literature, it becomes clear that cytokines can be a double-edged sword. They play a key role in child’s development, but they also can be harmful when a newborn has necrotizing enterocolitis (NEC), and they can theoretically affect the development of gluten intolerance (MohanKumar et al. 2017; Olivares et al. 2015; Rentea et al. 2017).

Depending on the lactation period and factors such as the course of pregnancy, gestational age, and vaginal or caesarean *delivery,* there are changes in milk composition and cytokine concentration, whose expression is dynamic (Chollet-Hinton et al. 2014). Cytokine concentration is also included in this biological rhythm. Morais et al. (2015) suggested that cytokines are characterized by chronobiological fluctuations. The study showed that the concentration of IL-6 was highest in colostrum in the diurnal phase and TNF-α also was in higher amount in colostrum compared to mature milk.

The amount of these compounds is higher at the beginning of lactation than in mature milk as a result of changes taking place in a woman’s body during pregnancy and childbirth. However, when complications such as pre-eclampsia occur, high cytokine levels in mother’s milk may persist up to 30 days postpartum. This may be a consequence of a still active inflammatory reaction (Erbağci et al. 2005). In milk from a mother with allergies, one can observe higher concentration of cytokines produced by lymphocytes Th2 (IL-4, IL-13, IL-5, IL-10) and lower TGF-β (Hrdý et al. 2012; Prokesová et al. 2006; Ragib et al. 2009; Zizka et al. 2007).

The higher concentrations of pro-inflammatory cytokines, where Th1 cytokines are predominant is observed in functional disorders of the mammary gland such as mastitis, particularly in women with systemic syndromes (Buescher and Hair 2001; Mizuno et al. 2012). Mastitis affects form 2 to 33% of nursing women and it is inflammation of breast tissue. This is a potentially serious illness, cause by milk stasis and infection (Angelopoulou et al. 2018) (Table 3). Depression, stress, or posttraumatic pain may also contribute to increased levels of pro-inflammatory cytokines (Kendall-Tackett 2007). Increased cytokine concentrations in milk (mainly IL-1β) correlate with the occurrence of jaundice in newborns, though the mechanism which is still unexplained (Apaydin et al. 2012; Zanardo et al. 2007). The extreme cases of overactive cytokine-induced inflammatory response may hypothetically contribute to sudden death of neonates. However, this has not been confirmed by enough cases (Vennemann et al. 2012). Cytokines are essential in the formation of the immune system, and they contribute to the mechanisms of many diseases. Disturbed balance between Th1 and Th2 can lead to long-lasting health consequences since the window between beneficial and damaging levels is very narrow. Crossing this limit may trigger pathophysiological mechanisms leading to immune dysfunctions (Zanardo et al. 2005). The absence of clear description of the consequences of higher/lower levels of cytokines in human milk is a result of the limitations of the research conducted, e.g., the lack of follow-up monitoring of the children as they grow, so many interpretations are still based on suppositions. The reason of the non-clear conclusion, obtained from research study are also a small number of sample, unknown volume of milk, non-constant collection times between studies, differences in population, and lack of standardized methods used to detect a cytokines.

**Table 3:** Example of study where cytokine were detected in milk and correlated with mastitis or allergy mother’s problem

Diseases	Samples	Investigated cytokines*	Comments	Literature
Clinical mastitis	*N* = 8 (women with clinical mastitis)	Selected pro-inflammatory cytokines (IL-6, IL-1β, TNF-α) Selected endogenous cytokines control molecules (sIL-6R, SIL-1RII, STNFRI)^1^	• TNF-α were evaluated in 6 from 8 samples, whereas IL-6 5/8 and Il = 1β 3/8.	Buescher and Hair 2001
	*N* = 17 Group A, body temperature was > 38.5 °C Group B, without systemic symptoms	IL-6^1^	• Level of IL-6 is higher in mastitic milk than milk from healthy mothers. If the women had a systemic symptoms, these differences are more significant.	Mizuno et al. 2012
Subclinical mastitis	*N* = 110 (56 from left breast and 54 form right breast collected from 44 healthy women)	25 cytokines IL-2, IL-2R, IL12p40/70, IL-15, IFN-α, IFN-γ, MIG, IP-10, IL-4,IL-5, IL-13,IL-7, IL-17, GMCSF, IL-10, EPO, IL-1RA, TNF-α, IL-6, IL-8, IL-1β, RANTES, EOTAXIN^2^	• Factors associated with inflammation (e.g., TNF-α, IL-6, Il-8, IL-17) were significantly increased in SCM samples. • Only IL-4 from Th2 in higher concentrations in SCM samples • The Th1/Th2 ratio; predominant elevation of cytokines that belong to the Th1 –lymphocyte	Tuaillon et al. 2017
	*N* = 108 Categorized as SCM (Na:K > 0.6); non-SCM (Na:K < 0.6)	IL-1β IL-6 IL-8 TNF-α^3^	• IL-8 and TNF-α where higher in SCM mothers (most notably in transitional milk)	Li et al. 2018
Allergy	Colostrum milk (3 days after delivery) Allergic (*n* = 15–44) and non-allergic mothers (*n* = 15–64)	IL-4, IL-6, IL-8, IL-10, _IL-12, IL-13, IFN-γ, TGF-β1, eotaxain, GRO-α, RANTES, TNF-α, EGF^1^	• Cytokines present in colostrum with high quantities (mediana > 100 pg/mL): IL-4, IL-5, IL-10, IL-13, INF-γ, TGF-β, TNF-α, MCP-1, GRO-α, EGF • Colostrum form allergy mothers: IL-5, IL-10, and IL-4; higher levels compared to healthy mothers	Zizka et al. 2007
	9 healthy mothers 11 allergic mothers	IL-2, IL-4, IL-8, IL-10, IL-13, IFN-γ, TGF-β1, EGF^1,4^	• Expression of IL-4, IL-13, and EGF was higher and levels of IFN-γ decreased in the colostral cells of allergic mothers compared to healthy.	Hrdý et al. 2012
	13 allergic mothers 9 healthy mothers Colostrum (3 days) Mature milk (1 month)	IL-10, TGF-β1^1^	• TGF-β concentration had a significant difference between colostrum and mature milk in milk from allergy mothers. • Compared to a mature milk form two groups of mothers, the TGF-β concentration was significantly lower in allergy mother’s milk. • No significant differences of IL-10 within the same group • 46% children form allergy mothers presented atopic dermatitis symptoms, none from controls (6-month observation)	Rigotti et al. 2006
	21 allergic 21 healthy mothers Colostrum (4 days) Mature milk (3, 6, 12 months)	IL-4, IL-5,IL-6,IL-10, IL-13. IFN-γ, TGF-β^1^	• The tendency to higher concentration of IL-4 and IL-10 in milk from allergy mothers • In mature milk, higher IL-4 concentration and different dynamic of IL-10 in allergy mother's milk	Prokesová et al. 2006

*Detected by following method:

^1^ELISA

^2^Multiplex microbeads assay

^3^MILLIPLEX MAP Human High Sensitivity Cytokine panel

^4^PCR

The procedure of cytokine identification and quantification must deal with a highly heterogeneous matrix as biological sample. Furthermore, cytokines are present in milk in low concentrations, so the method must be highly selective and specific. We need to keep in mind the high number of cytokines and how they work in a complex network of relationships (Liu et al. 2016).

The tests most commonly used for cytokine determination are the commercially available ELISA (Bryan et al. 2016; Hawkes et al. 2001; Zizka et al. 2007) and PCR (Hrdý et al. 2012). A disadvantage of immuno-enzymatic assays such as ELISA and ELISASPOT is the problem they have with rapid and accurate determination of multiple cytokines at the same time. A wider range of analytical tools is offered by immunological multiplex tests, e.g., BI-PRO^TM^ Human Cytokine 21-plex Assay (Bio-Rad Laboratories, Milan, Italy) (Kemp et al. 2015; Radillo et al. 2013) (Table 3). They consist of a bead on which specific capture antibodies are immobilized. This method is very sensitive and allows the researchers to determine analytes at low concentration levels (pg/ml). In addition, these tests also require smaller sample volumes (Keustermans et al. 2013). Cytokine detection was the subject of several reviews (Keustermans et al. 2013; Liu et al. 2016; Stenken and Poschenrieder 2015).

### Immunotoxicity of heavy metals

Metallic elements that are characterized by relatively high density and toxicity at low concentrations are known as heavy metals. These elements include cadmium, lead, mercury, and arsenic (Shaban et al. 2016). Heavy metals are external factors which can influence cytokine production. They are able to cross placenta and blood-brain barrier, and they can be present in women’s milk as well. This poses a potential risk for newborns (Rebelo and Caldas 2016).

The proven toxic effects of heavy metals on brain, kidney, and liver are particularly dangerous in situations when more than one metal is present. For instance, in a study carried out on laboratory animals (mice), simultaneous exposure to lead, cadmium, and mercury led to degradation of neurons in the brain. Addition of arsenic caused renal tubular necrosis (Cobbina et al. 2015). Heavy metal influences also on neurodegerative diseases as Alzheimer’s (Tan et al. 2014; Hussien et al. 2018).

**Fig. 5:**
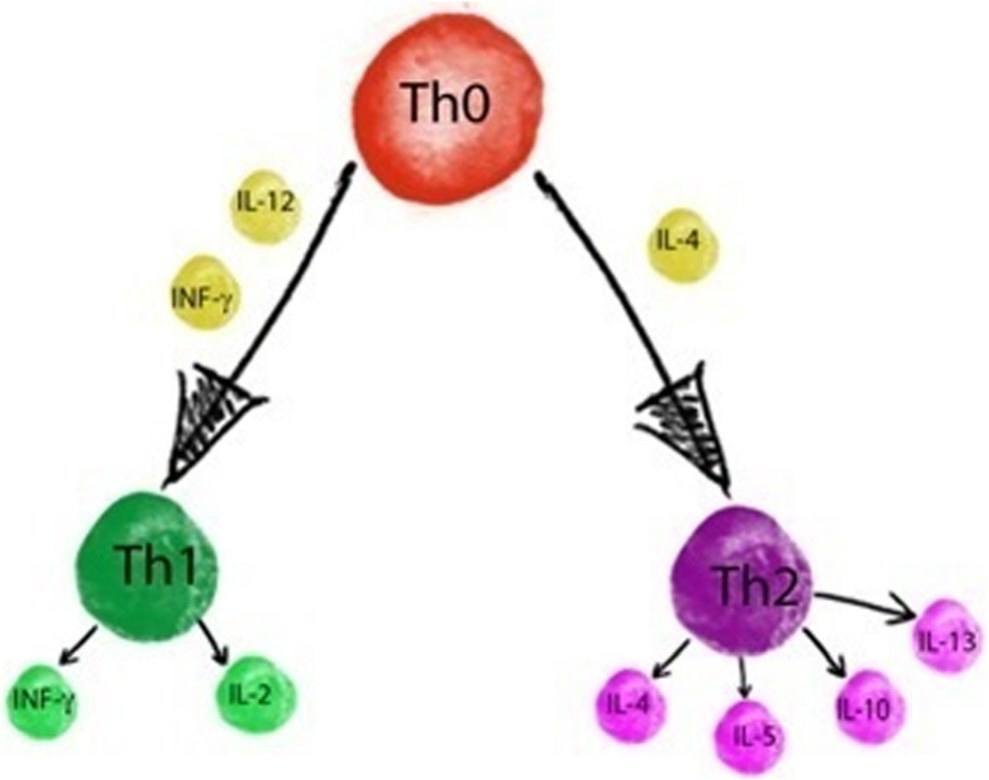
Production and interactions of cytokines

Immunotoxicity of heavy metals includes changes in the immune system (Rowley and Monestier 2005). Changes in cellular and humoral responses caused by metals may contribute to the development of autoimmune diseases, atopy, and allergies; they may disrupt sleep and also play a part in causing depression (Elenkov et al. 2005; Heo et al. 1997). These changes are reflected in the production of cytokines—hormone-like peptides, which are produced by lymphocytes Th (as a result of inflammatory action). Cytokines work in a complex networks (Elenkov et al. 2005). They are necessary for the initiation and regulation of the immune response (specific and non-specific), and they also influence the nature of such reactions (Kaiser et al. 2004). Even low concentrations of heavy metals can cause changes in cytokine productions (Låg et al. 2016). Lymphocytes Th1 produce interleukin 2 (IL-2), interferon gamma (IFNγ), and tumour necrosis factor-beta (TNF-β), while cytokines such as IL-4, IL-5, and IL-13 are produced by lymphocytes Th2. Both lymphocytes Th1 and Th2 produce IL-3, IL-6, IL-10, tumour necrosis factor-alpha (TNF-α), and granulocyte-macrophage colony-stimulating factor (*GM*-*CSF*) (Fig. 5) (Heo et al. 1996).

As a result of the presence of heavy metals, the production of cytokine Th1 may increase whereas the production of cytokine Th2 may be inhibited (or the other way round) (Krocova et al. 2000). In their research, Heo et al. (1996) showed that lead increased the amount of IL-4 produced, and decreased IFNγ. Disregulation occurs due to the dominance of cytokines produced by Th2. Krocova et al. (2000) also showed that production of cytokines Th2 was preferred during their study on the effects of lead and cadmium on mouse cells. A change in cytokine profile (Th1/Th2) can have dangerous consequences to fast and effective immune response. An example of such negative action was presented by Slovak scientists (Dvorožňáková et al. 2016), who showed that lead and cadmium inhibit the production of cytokines Th1. The metals suppress defense mechanisms and make it possible for an infection to spread rapidly, e.g., in the case of a parasitic infection. Not only the immune system is exposed to harmful effect on heavy metals and cytokine modulation. Cytokines play a crucial role in development of the central nervous system (CNS). Kasten-Jolly et al. (2011) conducted research on post-natal-day 21 mice, where Pb modulated IL-6, TGF-β1 and IL-18 protein expression in brain. Overexpression of IL-6 and TGF-β1 may harmfully affect neuronal growth and cell differentiation. Another heavy metal as mercury induced effect on neuroimmune signaling, mercury exposure cause increase in TNF-α expression in hippocampus and cerebellum (test in prairie voles) (Thomas Curtis et al. 2011). Interesting studies have been conducted by Gump et al. 2014. They showed that increasing blood Hg (9–11 children) was correlated with lower concentration of TNF-α in whole blood and shorter sleep duration.

There is no doubt that compounds like cytokines play a key role in proper functioning of the immune system, particularly a developing one. It is also known that any impairment of any function of the body can lead to irreversible damage caused by pathogens. Cytokines are essential to the immune response, and heavy metals can lead to cytokine production disorder and cause health problems. The hazard posed by heavy metals, including sources of exposure, mechanism of absorption, and metabolism of four metals—arsenic, mercury, cadmium, and lead—is described in more detail in “Heavy metals” section.

## Heavy metals

### Arsenic

**Fig. 6:**
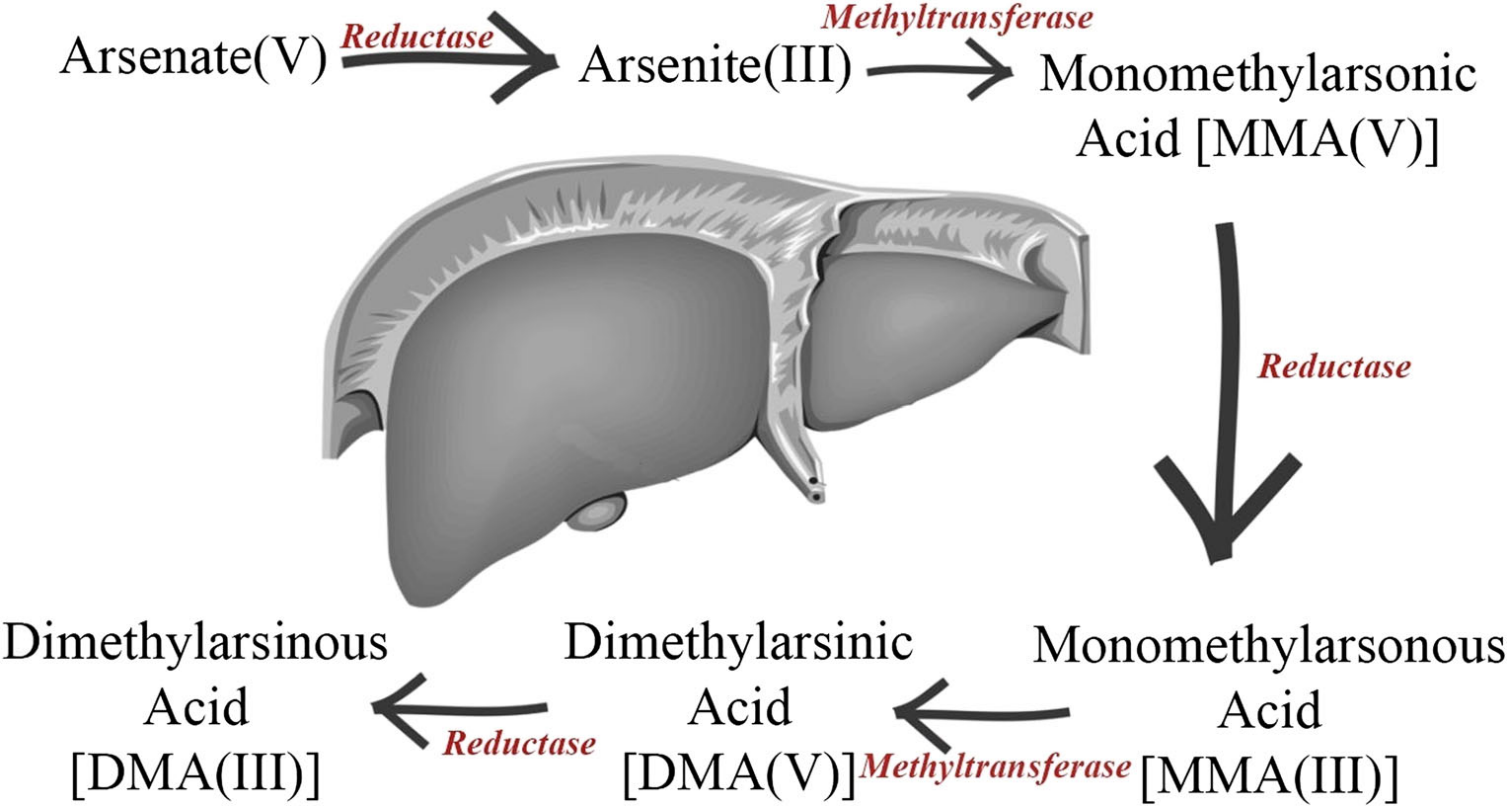
Metabolism of arsenic

Arsenic (As) exists in the environment in inorganic forms (iAs) as A^sIII^ and As^V^ and in such organic forms as monomethylarsenic (MMA) or dimethylarsenic (DMA), arsenobetaine, and arsenolipids (Rebelo and Caldas 2016). Inorganic forms of arsenic are soluble in water, whence the spread of arsenic in the environment. Consequently, this causes soil contamination and leads to accumulation of arsenic in food (rice and other grains and their products) (Cubadda et al. 2017; Ohno et al. 2007). In products of marine origin, arsenic is present in organic form (Taylor et al. 2017). Arsenic is rapidly absorbed by the digestive tract (As^III^ and As^V^). Arsenic in state V of oxidation reduces to III, followed by methylation using SAM (S-adenosylmethioninie) in the presence of GSH (glutathione). This leads to less toxic products such as DMA and MMA, which are excreted mainly with urine (Fig. 6). This is a detoxification process; however, in this pathway, reactive intermediates such as MMA^III^ and DMA^III^ may arise, which can have genotoxic effects (Beyersmann and Hartwig 2008; Gomez-Gaminero et al. 2001; Sattar et al. 2016; Vahter 2002). Exposure to arsenic during pregnancy is associated with the lower body of newborn child, weakening of cognitive functions, and even in extreme cases with fetal death (Carignan et al. 2015). The cross-sectional research (*n* = 190) revealed that exposure to arsenic can be associated with respiratory diseases such as asthma and with tachycardia (the detected concentrations of As were in the range of 36.6–82.7 μg/g creatinine) (Bortey-Sam et al. 2018).

In studies conducted on human milk (Table 4), no observed arsenic concentration would cause anxiety, even from mothers who lived near a contaminated area (for 187 samples tested, in 154 As was below LOD) (Sternowsky et al. 2002). However, it is worth it to pay attention to the research (Islam et al. 2014) where not only milk was analyzed but also urine from mothers and children. In contrast to low concentrations of arsenic in milk, in urine, its level was higher. It was suggested that exposure to this metal may be due to factors other than breastfeeding, for example, the presence of arsenic in water. It is *important to highlight* the fact *that* neonates who are breastfed are less exposed to arsenic than children who eat formula feed (as water is needed to prepare such milk) (Carignan et al. 2015; Castro et al. 2014; LaKind et al. 2001).

**Table 4:** Arsenic in human milk

Country	Lactation day (no. of samples)	Mean*/geometric mean**/(range μg/L)	Comments	Reference
Germany	Day 2 (18)	0.20** (0.15–1.1)	187 samples tested, in 154 As was below LOD (0.3 μg/L). Low concentration of arsenic in milk from mothers who live in a contaminated area suggests that breastfeeding does not contribute to the exposure of newborns to this metal.	(Sternowsky et al. 2002)
	Day 5 (93)	0.21 (0.15–2.5)		
	Day 15 (18)	0.16 (0.15–0.8)		
	Day 30 (11)	0.54 (0.15–2.8)		
	Day 45 (11)	0.19 (0.15–2.0)		
	Day 60 (12)	0.20 (0.15–0.9)		
	Day 75 (11)	0.16 (0.15–0.3)		
	Day 90 (13)	0.17 (0.15–0.8)		
Bangladesh	Month 1 (29)	1.12* (0.5–8.90)	In mother’s milk, the content of arsenic compared to the mother’s and baby’s urine was low (e.g., mean As content in milk:child urine:mother urine (μg/L) 1.12:157.8:18.1).	(Islam et al. 2014)
	Month 6 (25)	0.78 (0.5–2.32)		
	Month 9 (19)	0.70 (0.5–1.68)		
Chile (Arica, Santiago)	Arica (24), mine tailing deposition	0.36** (0.04–2.82)	In drinking water, concentration of arsenic was higher than in milk; as a result, children who are breastfed are less exposed.	(Castro et al. 2014)
	Santiago (11), control area	0.23** (0.08–0.61)		
Taiwan	Days 1–4	1.50	As lactation period progressed, the amount of arsenic in milk was decreasing.	(Chao et al. 2014)
	Days 5–10	0.68		
	Days 30–35	0.27		
	Days 60–65	0.16		
Sweden	Days 14–21 (60)	0.55* (0.041–4.6)	–	(Björklund et al. 2012)
Japan	Month 3 (9)	(0.18–4.20)	–	(Sakamoto et al. 2012)
Cyprus	50 samples	0.73* (0.03–1.97)	No significant correlation between moldy food consumption or the residential area	(Kunter et al. 2017)
Lebanon	74 nursing mothers (3–8 weeks of delivery)	*2.36 (0.08–11.32)	Arsenic was found in 63.51% of samples and this contamination was associated with cereal and fish intake.	(Bassil et al. 2018)

In the studies where the sample of milk was analyzed more than for one period of lactation, the arsenic concentrations were decreased. The higher mean concentration was presented in milk from Lebanon (Africa), this can be connected with drinking water contamination (Table 4).

### Mercury

Mercury occurs in the environment in organic and inorganic forms as well as in the elemental form (Hg^0^). The last one is found predominantly in the atmosphere. Water environment is rich in mercury in the second oxidation state (Hg^2+^). As a result of methylation of inorganic mercury, methylmercury is formed (Wong 2017). The main sources of mercury in milk include diet rich in fish (especially marine) (Grzunov Letinić et al. 2016), amalgam fillings (Drasch et al. 1998; Drexler and Schaller 1998; Grzunov Letinić et al. 2016), and residence in mining areas (Bose-O’Reilly et al. 2008). The findings of epidemiological studies (98 infertile female patients and a control group of 43) conducted by Maeda and co-workers (Maeda et al. 2019) suggest that methylmercury may affect female fertility.

**Fig. 7:**
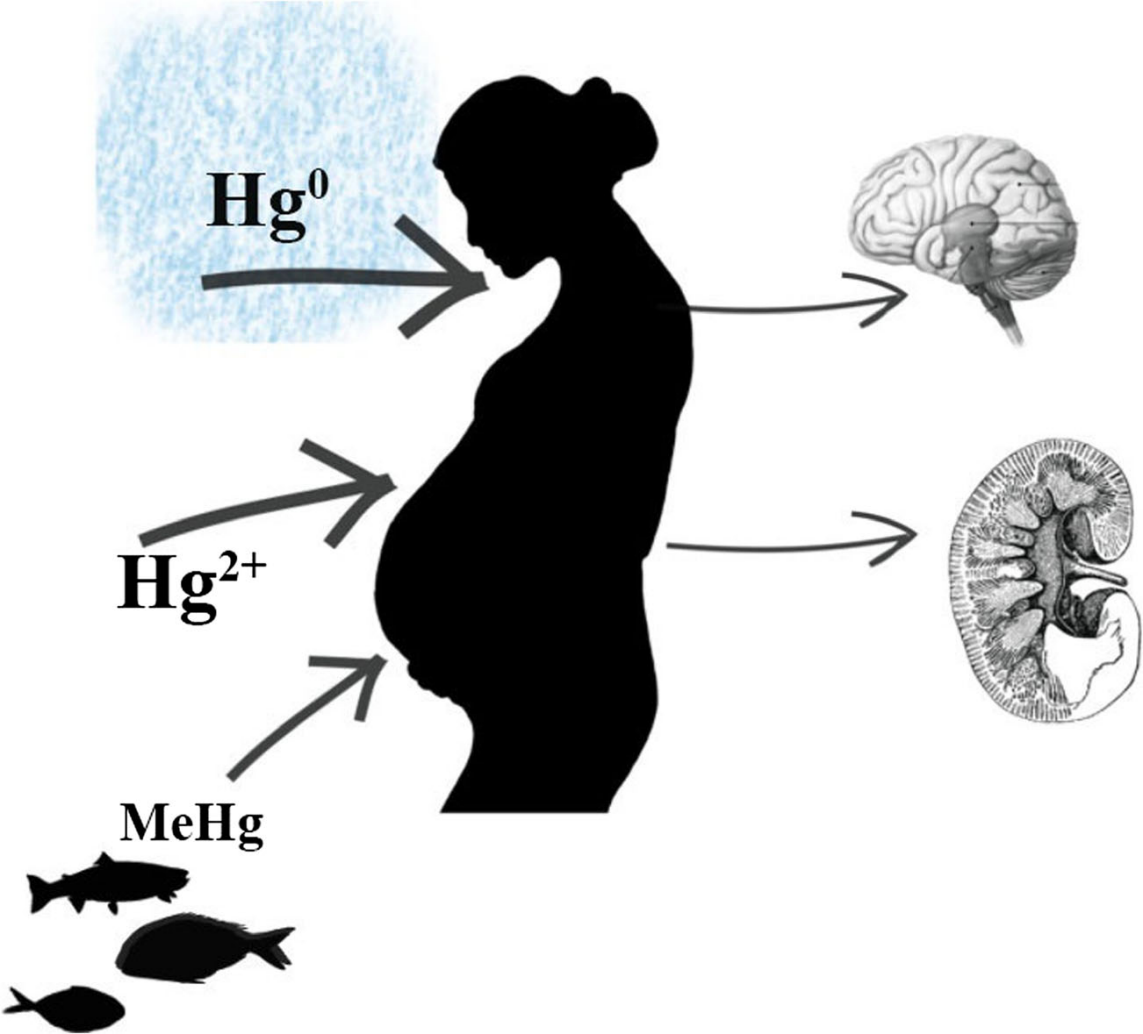
Mercury accumulation

Hg^0^ is absorbed into the body mainly via the respiratory track, and on a lesser scale via the digestive track or transdermally. After absorption, Hg oxidizes to Hg^2+^. To a large extent, mercury ions are deposited in the kidneys. The organic forms of mercury are very well absorbed by the digestive as well as respiratory track. After absorption, MeHg is quickly transported to tissues with high density of fat (Fig. 7). Ionic mercury is removed from the body with urine and feces, while MeHg is secreted with bile (Akerstrom et al. 2017). The high lipophilicity of the organic mercury leads to easy breakdown of the blood-brain barrier and facilitates its transport to placenta (Holmes et al. 2009). Exposure to Hg during lactation affects oxidative stress and can contribute to the pathogenesis of health problems. This is particularly dangerous during neuronal development (Al-Saleh et al. 2013). The results of milk analyses from different countries are presented in Table 5.

**Table 5:** Mercury in milk

Country	Lactation day (no. of samples)	Mean*/geometric mean**/(range μg/L)	Comments	Reference
Germany	Days 2–7 (70)	(0.2–6.86)	Presence of mercury was linked with the number of amalgam fillings the mother had. The average content was below 0.2 μg/L; when mother had 1–4 fillings, 0.57 μg/L; if more than 7, the content was 2.11 μg/L. Diet rich in fish also had an influence on mercury concentration in milk.	(Drasch et al. 1998)
	Week 1 (116)	1.37* (< 0.25–20.3)	Number of amalgam fillings in mother’s teeth and eating habits (fish diet) influence mercury concentration in milk. The concentration of mercury in milk after 2 months (mature milk) was lower than shortly after birth.	(Drexler and Schaller., 1998)
	Month 2 (84)	0.64 (0.25–11.7)		
Indonesia Tanzania Zimbabwe	Any day (46)	8.11* (< 1.0–149.60)	Area of residence where mercury is present may be a source of hazard. Mercury may be present in a woman’s milk at high concentrations, even if its content in her hair is low.	(Bose-O’Reilly et al. 2008)
Spain (Madrid)	Week 3 (100)	0.53** (0.03–2.63)	Concentration of Hg increased with number of amalgam fillings and amount of consumed fish and seafood.	(García-Esquinas et al. 2011)
Japan	Month 3 (9)	(0.28–0.77)	–	(Sakamoto et al. 2012)
Brazil (Brasilia)	Σ(147)		In the first three months after birth, mercury content in milk was not correlated with the amount of fish consumed by the mother. Intentional introduction of salmon into the diet (on day 75 of lactation) caused an increase in concentration of this metal in milk.	(Cunha et al. 2013)
	Day 15	THg 6.66*		
	Day 30	6.03		
	Day 45	6.02		
	Day 60	5.31		
	Day 74	6.01		
	Day 75	6.52		
	Day 76	7.29		
	Day 90	7.89		
Cyprus	50 samples	0 (0–0.01)	No significant correlation between moldy food consumption or the residential area	(Kunter et al. 2017)
Brazil	224 samples	THg 2.56* (< 0.76–8.40)	The limitation of this research was lack of information about time of sample collection, lack of food consumption information, or the number of amalgams of mothers. The levels of mercury including MeHg aren’t dangerous.	(Rebelo et al. 2017)
Korea Seoul (mega city) Anyaig (a residental city) Ansen (industrial complex) Jeju (mid-sized city)	207 samples	0.94* (0.08–5.66)	Mercury concentration was higher in milk form primipara mothers, and women over 30 years age, which are living in a big city. In the 15th day, sample levels of mercury was higher than in the 30-day breast milk sample. Mercury was detected in 100% sample.	(Park et al. 2018)
	Day 15	1.19		
	Day 30	0.79		

In the study in Brazil (2013) and Indonesia, Tanzania, and Zimbabwe, mercury was presented in the highest concentrations. However, in another research from Brazil, but from different regions, mercury concentration was much lower (Table 5).

### Lead and cadmium

**Fig. 8:**
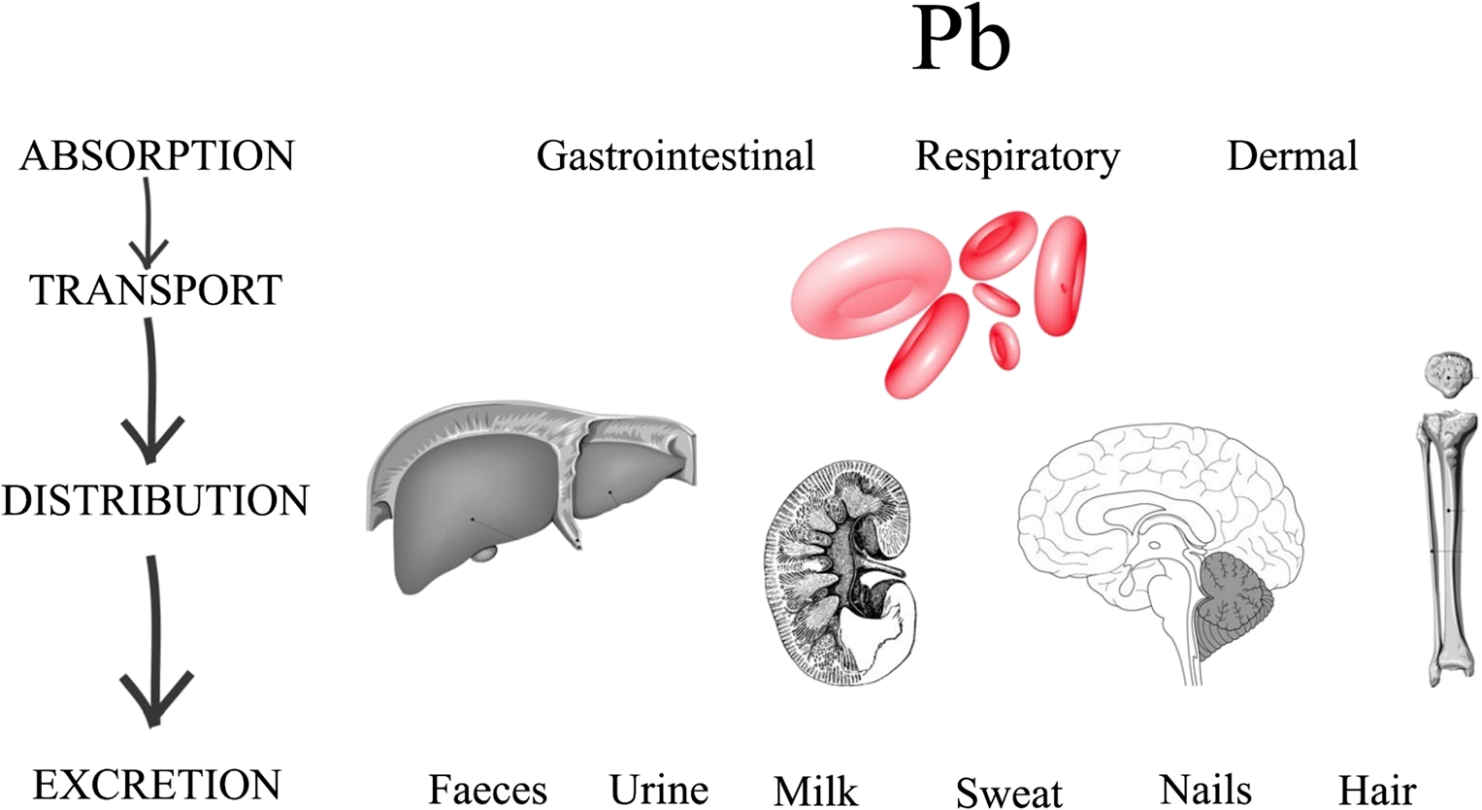
Lead absorption, distribution, and excretion

Among the metals prevalent in nature and dangerous for living organisms, lead also occupies a prominent position. Ninety percent of lead is accumulated in the bones (Gulson et al. 2003). This metal can get into organism in three ways: through the digestive and respiratory system and transdermally. It is transported by red blood cells into the liver and kidneys; the nervous system is also at risk. Lead is excreted from the body with urine, feces, and sweat; it also deposits in hair. Unfortunately, mother’s milk is one of the ways of eliminating this heavy metal from a female body (Babayigit et al. 2016; Wani et al. 2015) (Fig. 8). Lead contributes to the production of reactive oxygen in the body, which in turn contributes to the destruction of the existing molecules such as enzymes, proteins, and even DNA (Flora et al. 2012). Lead shows high affinity to the thiol group (-SH), which is involved in the destruction of proteins (Needleman 2004). Exposure to lead stems mainly from the place of residence, as milk from women living in urban areas contains higher concentrations of lead than the milk from women from rural areas (García-Esquinas et al. 2011; Leotsinidis et al. 2005). According to the World Health Organization (WHO 2010), the pediatric effects of Pb at various blood levels are developmental toxicity (10 μg/dL), increased nerve conduction velocity (20 μl/dL), decreased hemoglobin synthesis (40 μg/dL), and death when dosage exceeded 150 μg/dL. Moreover, in the blood of children from an industrialized area (*n* = 266), higher concentration of Pb in blood was detected (mean, 65.89 μg/L) than in the blood of children from a reference town (*n* = 264). Furthermore, this exposure can affect the nervous system and intelligence quotient scores, which was suggested in cross-sectional investigation about exposure of children to heavy metals (Pan et al. 2018). The presence of lead in milk does not necessarily result from mother’s direct exposure during pregnancy or lactation period. This heavy metal has a tendency to deposit in bones, where it remains for life. A proof of this is higher concentration of lead in milk of women aged over 30 (Chao et al. 2014). This compound may be released into body and milk as a result of bone resorption (changes during pregnancy and lactation) (Gulson et al. 2003).

**Fig. 9:**
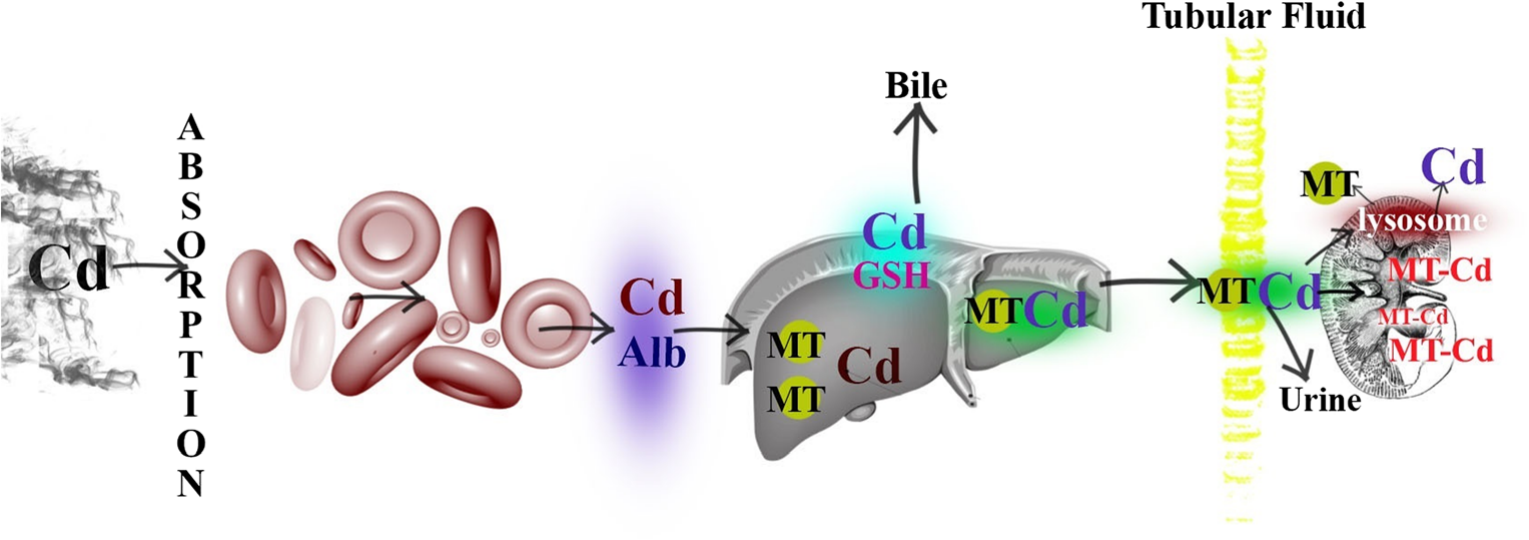
Absorption, distribution, and metabolism of cadmium

Smoking is also a source of exposure to lead (Grzunov Letinić et al. 2016) as well as to another heavy metal, cadmium (Cd) (Chao et al. 2014; García-Esquinas et al. 2011; Grzunov Letinić et al. 2016). The exposure to this metal is dependent on lifestyle, diet, and place of residence, and recorded values of daily intake range from 10 μg to more than 200 μg (Mezynska and Brzóska 2018).. Cadmium gets into the body mainly through respiratory track and less through digestive track. It has harmful impact on internal organs (Zalups and Ahmed 2003). After binding to proteins (albumin and metallothionein, MT), cadmium is transported with blood. The primary affected organ is liver (CdCl_2_), but kidneys are also exposed to long-term accumulation. This is a consequence of strong affinity to the MT protein (complex Cd-Mt). Cadmium is excreted from the body with feces and urine (Godt et al. 2006; Sarkar et al. 2013; Sinicropi et al. 2010; Waalkes 2000) (Fig. 9). Unfortunately, cadmium is one of the metals which are associated also with asthma prevalence; urinary concentrations of Cd in the control (*n* = 551) and case group (*n* = 551) were found to be 0.49 μg/g and 0.62 μg/g creatinine, respectively (Huang et al. 2016). Wang and co-workers (Wang et al. 2016) found that this metal may contribute to preterm birth. Their data (*n* = 3254) showed the serum Cd concentration with a range between 0.04 and 8.08 μg/L. Higher levels of cadmium were positively correlated with risk of preterm birth. The results of milk analyses from different countries are given in Table 6.

**Table 6:** Cadmium and lead in milk

Country	Lactation day (no. of samples)	Mean*/geometric mean**/(range μg/L)	Comments	Reference
Croatia	Day 4 Days 5–10 Days 20–30 (107)	Pb 2.4–10 1.9–12 1.7–7.2	Smoking is a source of lead and cadmium intake.	(Grzunov Letinić et al., 2016)
		Cd 0.6–1.4 0.58–1.4 0.59–1.6		
Spain	Week 3 (100)	Pb 15.65 **(12.92–18.72)	Concentration of lead increased with the amount of potatoes consumed. Amount of cadmium increased with frequency of smoking.	(García-Esquinas et al. 2011)
		Cd 1.31* (1.15–1.48)		
Greece	Day 3 (180)	Pb 0.48*	Women from urban areas were more exposed.	(Leotsinidis et al. 2005)
		Cd 0.19*		
Poland	323 milk samples	Pb 6.33*	Higher concentrations of heavy metals were determined in milk from smokers and older women (aged 30+).	(Winiarska-Mleczan 2014)
		Cd 2.11*		
Japan	Month 3 (9)	Pb (0.18–4.20)	–	Sakamoto et al. 2012
		Cd (0.40–1.80)		
Cyprus	50 samples	Pb 1.19* (0–4.91)	No significant correlation between moldy food consumption or the residential area	Kunter et al. 2017
		Cd 0.45* (0.12–0.80)		
Lebanon	74 samples	Pb 18.17* (1.38–62.61) Cd 0.87* (0.05–5.00)	Cadmium was detected in 40.54% of samples and was significantly associated random smoke exposure. Lead was detected in 67.61% of samples and contamination was correlated with residence near cultivation activities, smoking habits before pregnancy, and potato consumption.	Bassil et al. 2018
Korea Seoul (mega city) Anyaig (a residental city) Ansen (industrial complex) Jeju (mid-sized city)	207 samples Day 15 Day 30	Pb 8.79* 18.3 9.55	The highest level of lead was detected in breastmilk sample from a residential city in both sample dates (15 and 30 days) Lead concentration were not be correlated by age or smoking. Lead was detected in 77% of sample.	Park et al. 2018

In these two heavy metals, lead is predominant, which was detected in sample with higher concentration than cadmium (Table 6). This difference can be a result of strong accumulation of Pb on bone. Lactation may be associated with bone resorption, as a result to calcium demand for breastfed infants. The highest concentration was found in Lebanon and Spain. In both examples, the consumption of potato was associated with amount of lead.

Absorption of metals in newborn babies is more intense than in adults. Lack of mature defense system and still developing organs reduce the body’s ability to excrete toxic compounds with bile (Chao et al. 2014). Monitoring of milk from a single mother at different lactation stages (starting with the first days) reveals that concentration of heavy metals in milk shows a decreasing trend (Chao et al. 2014; Islam et al. 2014; Krachler et al. 1998; Leotsinidis et al. 2005) It is suggested that this trend is caused by the changes in the amount of milk protein that bind metals and fat content during the lactation (Sowers et al. 2002; Park et al. 2018) It is not possible to clearly evaluate factors contributing to and the degree of exposure to heavy metals. Recently conducted studies are focused on specific areas, where such factors may be different. Milk monitoring requires a sampling plan and a detailed interview regarding potential sources of exposure. Furthermore, it is desirable to analyze samples from more than one stage of lactation for each woman. In most cases metal analysis uses atomic absorption spectrometry (AAS) (Al-Saleh et al. 2013; Chao et al. 2014; Costopoulou et al. 2006; Drasch et al. 1998; Goudarzi et al. 2013; Islam et al. 2014; Winiarska-Mleczan 2014) and inductively coupled plasma mass spectrometry (ICP-MS) (Grzunov Letinić et al. 2016; Kunter et al. 2017).

## Breastfeeding: a threat or the gold standard of nutrition?

The problem of widespread environmental pollution and the ease with which it penetrates into human milk may lead to risks for the mother and newborn. From a scientific point of view, the double role of cytokines is very interesting. Their impaired secretion can lead to autoimmune diseases. Their presence in milk and long-term health consequences are still nor fully investigated. While their role is undeniable as the necessary immunomodulators, is breastfeeding the best food for a newborn? To answer this question, we should look at the benefits of breastfeeding (Andreas et al. 2015; Kulinich and Liu 2016; Lӧnnerdal 2016; Mandal et al. 2014; Nonqonierma and FlitzGerald 2015). Table 7 shows the main milk ingredients with their functions in the newborn’s body.

**Table 7:** Ingredients of human milk and their main functions

Ingredient	Functions
Proteins	• Nutritional (binding of essential ingredients, absorption through the intestinal mucosa) • Immunomodulatory and anti-inflammatory action • Anti-microbial effect • Normal bone formation • Growth promoters
Non-protein nitrogen	• Key role in cellular processes, i.e., changing enzymatic activity • Participation in the development, maturation, and repair of the digestive tract • Neurotransmitters
Lipids	• The largest source of energy • Formation of the nervous system incl. brain and spinal cord • Participation in neurobehavioral development • Correct retinal growth and visual acuity
Oligosaccharides	• Supporting growth of beneficial organisms (probiotics) • Nutritional microflora of the digestive tract • Protection against gastrointestinal infections • Prevention of diarrhea and respiratory infections

World Health Organization (WHO) recommends to exclusively breastfeed infants for 6 months at minimum. In many milk contamination studies, it is noted that the benefits of breastfeeding outweigh its potential risks (Chao et al. 2014; Islam et al. 2014; Leotsinidis et al. 2005). Formula feeds which are commercially available have less essential ingredients than natural milk. Furthermore, the former also may contain pollution as the water that is used to dissolve the powder may be contaminated. Consequently, formula feeding also can be a reason of imbalance of cytokine production (Akhtar et al. 2017; Castro et al. 2014; Chao et al. 2014; Dabeka et al. 2011; LaKind et al. 2001; Tripathi et al. 1999; Winkler et al. 2015).

## Conclusion

Organic and inorganic pollutants which are present in the environment can penetrate into living organisms. They are lipophilic and thus can accumulate in tissues, which can cause negative health consequences in the future. A newborn organism, which is still developing its organs and defense mechanisms, is particularly vulnerable. These compounds may get into a neonate’s body with mother’s milk. However, because human milk is the gold standard of nutrition and milk composition is unique, it is not recommended to give up this food. Furthermore, human milk as a non-invasive matrix is a tool used to analyze environmental pollution and serves also as a disease marker for the mother and child. In further research, attention should be paid to the consequences that can result from exposure to pollution, e.g., the effect of impurities on nutrients and immunomodulators as well as cytokine disregulation. The studies in this field are often limited by the sample size. It is important to develop a method of determination that will be both sensitive and efficient. Furthermore, it is necessary to conduct more coordinated research using an interdisciplinary approach to understand the role of environmental impurities such as PCBs and heavy metals in developmental process of an infant, especially with regard to the role of cytokines. For these reasons, the crucial and the first point is to propose a specific analytical tool (i.e., innovative adsorbents as molecularly imprinted polymers (MIPs) and sensor for analysis of these compounds in human milk. The combination of new tools with modern instrumentation techniques will allow quick and reliable assessment of both the level of milk contamination and the content of cytokines. Moreover, it will be of critical importance to conduct a cohort research with a representative number of samples. The integration of such different fields as chemistry, biology, and medicine with statistical analysis can help understand the influence of environmental agents on human milk quality and consequently on children’s health.

## References

[CR1] Agarwal S, Karmaus W, David S, Gangur V (2011). Immune markers in breast milk and fetal and maternal body fluids: a systematic review of perinatal concentrations. J Hum Lact.

[CR2] Akerstrom M, Barregard L, Lundh T, Sallsten G (2017). Relationship between mercury in kidney, blood, and urine in environmentally exposed individuals, and implications for biomonitoring. Toxicol Appl Pharmacol.

[CR3] Akhtar S, Shahzad MA, Yoo SH, Ismail A, Hameed A, Ismail T, Riaz M (2017). Determination of aflatoxin M1 and heavy metals in infant formula milk brands available in Pakistani markets. Korean J Food Sci Anim Resour.

[CR4] Al-Saleh I, Abduljabbar M, Al-Rougi R, Elkhatib R, Alshabbaheen A, Shinwari N (2013). Mercury (Hg) exposure in breast-fed infants and their mothers and the evidence of oxidative stress. Biol Trace Elem Res.

[CR5] Amsen D, Splianakis CG, Flavell RA (2009). How are T(H)1 and T(H)2 effector cells made?. Curr Opin Immunol.

[CR6] Andersson PL, Haglund P, Tysklind M (1997). The internal barriers of rotation for the 209 polychlorinated biphenyls. Environ Sci Pollut Res Int.

[CR7] Andersson PL, Haglund P, Tysklind M (1997). Ultraviolet absorption spectra of all 209 polychlorinated biphenyls evaluated by principal component analysis. Fresenius J Anal Chem.

[CR8] Andreas NJ, Kampmann B, Mehring Le-Doare K (2015). Human breast milk: a review on its composition and bioactivity. Early Hum Dev.

[CR9] Angelopoulou A, Field D, Ryan CA, Stanton C, Hill C, Ross RP (2018). The microbiology and treatment of human mastitis. Med Microbiol Immunol.

[CR10] Antignac JP, Main KM, Virtanen HE, Boquien CY, Marchand P, Venisseau A, Guiffard I, Bichon E, Wohlfahrt-Veje C, Legrand A, Boscher C, Skakkebæk NE, Toppari J, Le Bizec B (2016). Contru-specific chemical signatures of persistent organic pollutants (POPs) in breast milk in French, Danish and Finnish women. Environ Poll.

[CR11] Apaydin K, Ermis B, Arasli M, Tekin I, Ankarali H (2012). Cytokines in human milk and late-onset breast milk jaundice. Pediatr Int.

[CR12] Arai K, Lee F, Miyajima A, Miyatake S, Arai N, Yokota T (1990). Cytokines: coordinators of immune and inflammatory responses. Annu Rev Biochem.

[CR13] Asamoah A, Essumang DK, Muff J, Kucheryavskiy SV, Søgaard EG (2018). Assessment of PCBs and exposure risk to infants in breast milk of primiparae and multiparae mothers in an electronic waste hot spot and non-hot spot areas in Ghana. Sci Total Environ.

[CR14] Baars AJ, Bakker MI, Baumann RA, Boon PE, Freijer JI, Hoogenboom LA, Hoogerbrugge R, van Klaveren JD, Liem AK, Traaq WA, de Vries J (2004). Dioxin, dioxin-like PCBs and non-dioxin-like PCBs in foodstuffs: occurrence and dietary intake in The Netherlands. Toxicol Lett.

[CR15] Babayigit A, Ethirajan A, Muller M, Conings B (2016). Toxicity of organometal halide perovskite solar cells. Nat Mater.

[CR16] Bahadar H, Abdollahi M, Maqbool F, Baeeri M, Niaz K (2015). Mechanistic overview of immune modulatory effects of environmental toxicants. Inflamm Allergy Drug Targets.

[CR17] Bányiová K, Černá M, Mikeš O, Komprdová K, Sharma A, Gyalpo T, Čupr P, Scheringer M (2017). Long-term time trends in human intake of POPs in the Czech Republic indicate a need for continuous monitoring. Environ Int.

[CR18] Barrow CJ, Nolan C, Holub BJ (2009). Bioequivalence of encapsulated and microencapsulated fish-oil supplementation. J Funct Foods.

[CR19] Bassil M, Daou F, Hassan H, Yamani O, Kharma JA, Atteh Z, Elaridi J (2018). Lead, cadmium and arsenic in human milk and their socio-demographic and lifestyle determinants in Lebanon. Chemosphere.

[CR20] Bencko V, Cerná M, Jech L, Smίd J (2004). Exposure of breast-fed children in the Czech Republic to PCDDs, PCDFs, and dioxin-like PCBs. Environ Toxicol Pharmacol.

[CR21] Benjamin S, Masai E, Kamimura N, Takahashi K, Anderson R, Faisal PA (2017). Phthalates impact human health: epidemiological evidences and plausible mechanism of action. J Hazard Mater.

[CR22] Bernsten HF, Fonnum F, Walaas SI, Bogen IL (2016). Low-chlorinated non-dioxin-like polychlorinated biphenyls present in blood and breast milk induce higher levels of reactive oxygen species in neutrophil granulocytes than high-chlorinated congeners. Basic Clin Pharmacol.

[CR23] Beyersmann D, Hartwig A (2008). Carcinogenic metal compounds: recent insight into molecular and cellular mechanisms. Arch Toxicol.

[CR24] Björklund KL, Vahter M, Palm B, Grandér M, Lignell S, Berglund M (2012). Metals and trace element concentrations in breast milk of first time healthy mothers: a biological monitoring study. Environ Health.

[CR25] Bode L (2012). Human milk oligosaccharides: every baby needs a sugar mama. Glycobiology.

[CR26] Bortey-Sam N, Ikenaka Y, Akoto O, Nakayama SMM, Asante AK, Baidoo E, Obirikorang C, Mizukawa H, Ishizuka M (2018). Association between human exposure to heavy metals/metalloid and occurrences of respiratory diseases, lipid peroxidation and DNA damage in Kumasi, Ghana. Environ Pollut.

[CR27] Bose-O’Reilly S, Lettmeier B, Roider G, Siebert U, Drasch G (2008). Mercury in breast milk - a health hazard for infants in gold mining areas?. Int J Hyg Environ Health.

[CR28] Braun JM, Kalkbrenner AE, Calafat AM, Yolton K, Ye X, Dietrich KN (2011). Impact of early-life bisphenol A exposure on behavior and executive function in children. Pediatrics.

[CR29] Bryan DL, Forsyth KD, Gibson RA, Hawkes JS (2016). Interleukin-2 in human milk: a potential modulator of lymphocyte development in the breastfed infant. Cytokine.

[CR30] Bu Q, Macleod M, Wong F, Toms L, Mueller J, Yu G (2015). Historical intake and elimination of polychlorinated biphenyls and organochlorine pesticides by the Australian population reconstructed from biomonitoring data. Environ Int.

[CR31] Buescher ES, Hair PS (2001). Human milk anti-inflammatory component contents during acute mastitis. Cell Immunol.

[CR32] Carignan CC, Cottingham KL, Jackson BP, Farzan SF, Gandolfi AJ, Punshon T, Folt CL, Karagas MR (2015). Estimated exposure to arsenic in breastfed and formula-fed infants in a United States cohort. Environ Health Perspect.

[CR33] Caspersen IH, Aase H, Biele G, Brantsaeter AL, Haugen M, Kvalem HE, Skogan AH, Zeiner P, Alexander J, Meltzer HM, Knutsen HK (2016). The influence of maternal dietary exposure to dioxins and PCBs during pregnancy on ADHD symptoms and cognitive functions in Norwegian preschool children. Environ Int.

[CR34] Caspersen IH, Haugen M, Schjolberg S, Vejrup K, Knutsen HK, Branstsaeter AL, Meltzer HM, Alexander J, Magnus P, Kvalem HE (2016). Maternal dietary exposure to dioxins and polychlorinated biphenyl (PCBs) is associated with language delay in 3 year old Norwegian children. Environ Int.

[CR35] Castro F, Harari F, LIanos M, Vahter M, Ronco AM (2014). Maternal-child transfer of essential and toxic elements through breast milk in a mine-waste polluted area. Am J Perinatol.

[CR36] Čechová E, Scheringer M, Seifertová M, Mikeš O, Kroupová K, Kuta J, Forns J, Eggesbø M, Quaak I, de Cock M, van de Bor M, Henrieta Patayová H, Palkovičová Murínová L, Kočan A (2017). Developmental neurotoxicants in human milk: comparison of levels and intakes in three European countries. Sci Total Environ.

[CR37] Černá M, Bencko V, Brabec M, Šmίd J, Kesková A, Jech L (2010). Exposure assessment of breast-fed infants in the Czech Republic to indicator PCBs and selected chlorinated pesticides. Area-related differences. Chemosphere.

[CR38] Chao HH, Guo CH, Huang CB, Chen PC, Li HC, Hsiung DY, Chou YK (2014). Arsenic, cadmium, lead, and aluminium concentrations in human milk at early stages of lactation. Pediatr Neonatol.

[CR39] Chen Y, Wang X, Li Y, Toms L-ML, Gallen M, Hearn L, Aylward LL, McLachlan MS, Sly PD, Mueller JF (2015). Persistent organic pollutants in matched breast milk and infant faeces samples. Chemosphere.

[CR40] Chollet-Hinton LS, Stuebe AM, Casbas-Hernandez P, Chetwynd E, Troester MA (2014). Temporal trends in the inflammatory cytokine profile of human breastmilk. Breastfeed Med.

[CR41] Chovancová J, Čonka K, Kočan A, Sejáková ZS (2011). PCDD, PCDF, PCB and PBDE concentrations in breast milk of mothers residing in selected areas of Slovakia. Chemosphere.

[CR42] Cobbina SJ, Chen Y, Zhou Z, Wu X, Zhao T, Zhang Z, Feng W, Wang W, Li Q, Wu X, Yang L (2015). Toxicity assessment due to sub-chronic exposure to individual and mixtures of four toxic heavy metals. J Hazard Mater.

[CR43] Costopoulou D, Vassiliadou I, Papadopoulos A, Makropoulos V, Leondiadis L (2006). Levels of dioxins, furans and PCBs in human serum and milk of people living in Greece. Chemosphere.

[CR44] Cubadda F, Jackson BP, Cottingham KL, Van Horne YO, Kurzius-Spencer M (2017). Human exposure to dietary inorganic arsenic and other arsenic species: State of knowledge, gaps and uncertainties. Sci. Total Environ.

[CR45] Cunha LR, Costa TH, Caldas ED (2013). Mercury concentration in breast milk and infant exposure assessment during the first 90 days of lactation in a midwestern region of Brazil. Biol Trace Elem Res.

[CR46] Dabeka R, Fouquet A, Belisle S, Turcotte S (2011). Lead, cadmium and aluminum in Canadian infant formulae, oral electrolytes and glucose solutions. Food Addit Contam Part A Chem Anal Control Expo Risk Assess.

[CR47] Deng B, Zhang J, Zhang L, Jiang Y, Zhou J, Fang D, Zhang H, Huang H (2012). Levels and profiles of PCDD/Fs, PCBs in mothers’ milk in Shenzhen of China: estimation of breast-fed infants’ intakes. Environ Int.

[CR48] Dinarello CA (2007). Historical insights into cytokines. Eur J Immunol.

[CR49] Dmitrovic J, Chan SC (2002). Determination of polychlorinated biphenyl congeners in human milk by gas chromatography–negative chemical ionization mass spectrometry after sample clean-up by solid-phase extraction. J Chromatogr B Anal Technol Biomed Life Sci.

[CR50] Drasch G, Aigner S, Roider G, Staiger F, Lipowsky G (1998). Mercury in human colostrum and early breast milk. its dependence on dental amalgam and other factors. J Trace Elem Med Biol.

[CR51] Drexler H, Schaller KH (1998). The mercury concentration in breast milk resulting from amalgam fillings and dietary habits. Environ Res.

[CR52] Duijts L, Jaddoe VW, Hofman A, Moll HA (2010). Prolonged and exclusive breastfeeding reduces the risk of infectious diseases in infancy. Pediatrics.

[CR53] Dvorožňáková E, Dvorožňáková M, Šoltys J (2016). Heavy metal intoxication compromises the host cytokine response in Ascaris Suum model infection. Helminthologia.

[CR54] Elenkov IJ, Iezzoni DG, Daly A, Harris AG, Chrousos GP (2005). Cytokine dysregulation, inflammation and well-being. Neuroimmunomodulation.

[CR55] Emmett PM, Rogers IS (1997). Properties of human milk and their relationship with maternal nutrition. Early Hum Dev.

[CR56] Ennaceur S, Ridha D, Marcos R (2008). Genotoxicity of the organochlorine pesticides 1,1-dichloro-2,2-3 bis(p-chlorophenyl)ethylene (DDE) and hexachlorobenzene (HCB) in cultured human lymphocytes. Chemosphere.

[CR57] Erbağci AB, Cekmen MB, Balat O, Balat A, Aksoy F, Tarakçioğlu M (2005). Persistency of high proinflammatory cytokine levels from colostrum to mature milk in preeclampsia. Clin Biochem.

[CR58] Erickson MD, Kaley RGII (2011). Applications of polychlorinated biphenyls. Environ Sci Pollut Res.

[CR59] Eskenazi B, Marks AR, Bradman A, Fenster L, Johnson C, Barr DB, Jewell NP (2006). In utero exposure to dichlorodiphenyltrichloroethane (DDT) and dichlorodiphenyldichloroethylene (DDE) and neurodevelopment among young Mexican American children. Pediatrics.

[CR60] Faroon O, Keith L, Smith-Simon C, De Rosa C (2003). Polychlorinated biphenyls. Human health aspects. Concise International Chemical Assessment Document 55.

[CR61] Fernandez AR, Rose M, White S, Mortimer DN, Gem M (2006). Dioxins and polychlorinated biphenyls (PCBs) in fish oil dietary supplements: Occurrence and human exposure in the UK. Food Addit Contam.

[CR62] Flora G, Gupta D, Tiwari A (2012). Toxicity of lead: a review with recent updates. Interdiscip Toxicol.

[CR63] Gao X, McMahnon RJ, Woo JG, Davidson BS, Morrow AL, Zhang Q (2012). Temporal changes in milk proteomes reveal developing milk functions. J Proteome Res.

[CR64] García-Esquinas E, Pérez-Gómez B, Fernández MA, Pérez-Meixeira AM, Gil E, de Paz C, Iriso A, Sanz JC, Astray J, Cisneros M, de Santos A, Asensio A, García-Sagredo JM, García JF, Vioque J, Pollán M, López-Abente G, González MJ, Martínez M, Bohigas PA, Pasto R, Aragonés N (2011). Mercury, lead and cadmium in human milk in relation to diet, lifestyle habits and sociodemographic variables in Madrid (Spain). Chemosphere.

[CR65] Garofalo R (2010). Cytokines in human milk. J Pediatr.

[CR66] Gascon M, Morales E, Sunyer J, Vrijheid M (2013). Effects of persistent organic pollutants on the developing respiratory and immune systems: a systematic review. Environ Int.

[CR67] Gevao B, Hamilton-Taylor J, Jones KC (1998). Polychlorinated biphenyl and polycyclic aromatic hydrocarbon deposition to and exchange at the air-water interface of Esthwaite Water, a small lake in Cumbria, UK. Environ Pollut.

[CR68] Godt J, Scheidig F, Grosse-Siestrup C, Esche V, Brandenburg P, Reich A, Groneberg DA (2006). The toxicity of cadmium and resulting hazards for human health. J Occup Med Toxicol.

[CR69] Gomez-Gallego C, Garcia-Mantrana I, Salminen S, Collado MC (2016). The human milk microbiome and factors influencing its composition and activity. Semin Fetal Neonatal Med.

[CR70] Gomez-Gaminero A, Howe P, Hughes M, Kenyon E, Lewis DR, Ng JC, Aitio A, Becking G (2001). Environmental heath criteria 224 arsenic and arsenic compounds.

[CR71] Goudarzi MA, Parsaei P, Nayebpour F, Rahimi E (2013). Determination of mercury, cadmium and lead in human milk in Iran. Toxicol Ind Health.

[CR72] Grandjean P, Landrigan PJ (2006). Developmental neurotoxicity of industrial chemicals. Lencet Neurol.

[CR73] Grandjean P, Landrigan PJ (2014). Neurobehavioral effects of developmental toxicity. Lancet Neurol.

[CR74] Granum B, Haug LS, Namork E, Stølevik SB, Thomsen C, Aaberge IS, Van Loversen H, Løvik M, Nygaard UC (2013). Pre-natal exposure to perfluroalkyl substances may be associated with altered vaccine antibody levels and immune-related health outcomes in early childhood. Environ Health Perspect.

[CR75] Grzunov Letinić J, Matek Sarić M, Piasek M, Jurasović J, Varnai VM, Sulimanec Grgec A, Orct T (2016). Use of human milk in the assessment of toxic metal exposure and essential element status in breastfeeding women and their infants in coastal Croatia. J Trace Elem Med Biol.

[CR76] Gulson BL, Mizon KJ, Korsch MJ, Palmer JM, Donnelly JB (2003). Mobilization of lead from human bone tissue during pregnancy and lactation-a summary of long-term research. Sci Total Environ.

[CR77] Gump BB, Gabrikova E, Bendinskas K, Dumas AK, Palamer CD, Parsons PJ, MacKenzie JA (2014). Low-level mercury in children: associations with sleep duration and cytokines TNF-α and IL-6. Environ Res.

[CR78] Hansen S, Strøm M, Olsen SF, Maslova E, Rantakokko P, Kiviranta H, Rytter D, Bech BH, Hansen LV, Halldorsson TI (2014). Maternal concentration of persistent organochlorine pollutants and the risk of asthma in offspring. Results from a prospective cohort with 20 years of follow-up. Environ Health Perpect.

[CR79] Hassine SB, Ameur WB, Gandoura N, Driss MR (2012). Determination of chlorinated pesticides, polychlorinated biphenyls, and polybrominated diphenyl ethers in human milk from Bizerte (Tunisia) in 2010. Chemosphere.

[CR80] Hawkes JS, Bryan DL, Neumann MA, Makrides M, Gibson RA (2001). Transforming growth factor beta in human milk does not change in response to modest intakes of docosahexaenoic acid. Lipids.

[CR81] Heo Y, Parsons PJ, Lawrence DA (1996). Lead differentially modifies cytokine production in vitro and in vivo. Toxicol Appl Pharmacol.

[CR82] Heo Y, Lee WT, Lawrence DA (1997). In vivo the environmental pollutants lead and mercury induce oligoclonal T cell responses skewed toward type-2 reactivities. Cell Immunol.

[CR83] Holmes P, James KA, Levy LS (2009). Is low-level environmental mercury exposure of concern to human health?. Sci Total Environ.

[CR84] Hrdý J, Novotná O, Kocourková I, Prokešová L (2012). Cytokine expression in the colostral cells of healthy and allergic mothers. Folia Microbiol (Praha).

[CR85] Huang H, Xie J, Cui X, Zhou Y, Wu X, Lu W, Shen Y, Yuan J, Chen W (2016). Association between concentrations of metal in urine and adult asthma: a case-control study in Wuhan, China. PLoS One.

[CR86] Hussien HM, Abd-Elmeied A, Ghareeb DA, Hafez HS, Ahmed HEA, El-Moneam NA (2018). Neuroprotective effects of berberine against environmental heavy metals-induced neurotoxixity and Alzheimer’s-like disease in rats. Food Chem Toxicol.

[CR87] Le Huёrou-Luron I, Blat S, Boudry G (2010). Breast-υ, formula-feeding: impacts on the digestive tract and immediate and long-term health effects. Nutr Res Rev.

[CR88] Islam MR, Attia J, Alauddin M, McEvoy M, McElduff P, Slater C, Islam MM, Akhter A, d’Este C, Peel R, Akter S, Smith W, Begg S, Milton AH (2014). Availability of arsenic in human milk in women and its correlation with arsenic in urine of breastfed children living in arsenic contaminated areas in Bangladesh. Environ Health.

[CR89] Kaiser P, Rothwell L, Avery S, Balu S (2004). Evolution of the interleukins. Dev Comp Immunol.

[CR90] Kasten-Jolly J, Heo Y, Lawrence DA (2011). Central nervous system cytokine gene expression: modulation by lead. J Biochem Mol Toxicol.

[CR91] Katsikantami I, Sifakis S, Tzatzarakis M, Vakonaki E, Kalantzi O-I, Tsatsakis A, Rizos AK (2016). A global assessment of phthalates burden and related links to health effects. Environ Int.

[CR92] Kemp TJ, Castro FA, Gao YT, Hildesheim A, Noqueira L, Wang BS, Sun L, Shelton G, Pfeiffer RM, Pinto LA, Koshiol J (2015). Application of multiplex arrays for cytokine and chemokine profiling of bile. Cytokine.

[CR93] Kendall-Tackett K (2007). A new paradigm for depression in new mothers: the central role of inflammation and how breastfeeding and anti-inflammatory treatments protect maternal mental health. Int Breast J.

[CR94] Keustermans GC, Hoeks SB, Meerding JM, Prakken BJ, de Jager W (2013). Cytokine assays: an assessment of the preparation and treatment of blood and tissue samples. Methods.

[CR95] Kim S, Eom S, Kim H-J, Lee JJ, Choi G, Choi S, Kim S, Kim SY, Cho G, Kim YD, Suh E, Kim SK, Kim S, Kim G-H, Moon H-B, Park J, Kim S, Choi K, Eun S-H (2018). Association between maternal exposure to major phthalates, heavy metals, and persistent organic pollutants, and the neurodevelopmental performances of their children at 1 to 2 years of age- CHECK cohort study. Sci Total Environ.

[CR96] Klinčić D, Herceg Romanić S, Brčić Karačonji I, Matek Sarić M, Grzunov Letinić J, Brajenović N (2016). Organochlorine pesticides and PCBs (including dl-PCBs) in human milk samples collected from multiparae from Croatia and comparison with primiparae. Environ Toxicol Pharmacol.

[CR97] Krachler M, Li FS, Rossipal E, Irqolic KJ (1998). Changes in the concentrations of trace elements in human milk during lactation. J Trace Elem Med Biol.

[CR98] Krocova Z, Macela A, Kroca M, Hernychova L (2000). The immunomodulatory effect(s) of lead and cadmium on the cells of immune system in vitro. Toxicol in Vitro.

[CR99] Kulinich A, Liu L (2016). Human milk oligosaccharides: the role in the fine-tuning of innate immune responses. Carbohydr Res.

[CR100] Kunter İ, Hürer N, Gülcan HO, Öztürk B, Doğan İ, Şahin G (2017). Assessment of aflatoxin M1 and heavy metal levels in mothers breast milk in Famagusta. Cyprus Biol Trace Elem Res.

[CR101] Låg M, Øvrevik J, Totlandsdal AI, Lilleaas EM, Thormodsæter A, Holme JA, Schwarze PE, Refsnes M (2016). Air pollution-related metals induce differential cytokine responses in bronchial epithelial cells. Toxicol In Vitro.

[CR102] LaKind JS, Berlin CM, Naiman DQ (2001). Infant exposure to chemicals in breast milk in the United States: what we need to learn from a breast milk monitoring program. Environ Health Perspect.

[CR103] Leotsinidis M, Alexpoulos A, Kostopoulou-Farri E (2005). Toxic and essential trace elements in human milk from Greek lactating women: association with dietary habits and other factors. Chemosphere.

[CR104] Li C, Solomons NW, Scott ME, Koski KG (2018). Subclinical mastitis (SCM) and proinflammatory cytokines are associated with mineral and trace element concentrations in human breast milk. J Trace Elem Med Biol.

[CR105] Lignell S, Aune M, Darnerud PO, Hanberg A, Larsson SC, Glynn A (2013). Prenatal exposure to polychlorinated biphenyls (PCBs) and polybrominated diphenyl ethers (PBDEs) may influence birth weight among infants in a Swedish cohort with background exposure: a cross-sectional study. Environ. Health.

[CR106] Lin Y, Feng C, Xu Q, Lu D, Qiu X, Jin Y, Wang G, Wang D, She J, Zhou Z (2016). A validated method for rapid determination of dibenzo-p-dioxins/furans (PCDD/Fs), polybrominated diphenyl ethers (PBDEs) and polychlorinated biphenyls (PCBs) in human milk: focus on utility of tandem solid phase extraction (SPE) cleanup. Anal Bioanal Chem.

[CR107] Liu G, Qi M, Hutchinson MR, Yang G, Goldys EM (2016). Recent advances in cytokine detection by immunosensing. Biosens Bioelectron.

[CR108] Lӧnnerdal B (2016). Bioactive proteins in human milk: health, nutrition, and implications for infant formulas. J Pediatr.

[CR109] Lopes BR, Barreiro JC, Cass QB (2016). Bioanalytical challenge: a review of environmental and pharmaceuticals contaminants in human milk. JPharm Biomed Anal.

[CR110] Luzardo OP, Ruiz-Suárez N, Amleida-González M, Henίquez-Hernádez LA, Zumbado M, Boada LD (2013). Multi-residue method for the determination of 57 persistent organic pollutants in human milk and colostrum using a QuEChERS-based extraction procedure. Anal Bioanal Chem.

[CR111] Maeda E, Murata K, Kumazawa Y, Sato W, Shirasawa H, Iwasawa T, Izumo K, Tatsuta N, Sakamoto M, Terada Y (2019). Associations of environmental exposures to methylmercury and selenium with female infertility: a case-control study. Environ Res.

[CR112] Maervoet J, Vermeir G, Covaci A, Van Larebeke N, Koppen G, Schoeters G, Nelen V, Bayens W, Schepens P, Viaene MK (2007). Association of thyroid hormone concentrations with levels of organochlorine compounds in cord blood of neonates. Environ Health Perspect.

[CR113] Mamontova EA, Tarasova EN, Mamontov AA (2017). PCBs and OCPs in human milk in Eastern Siberia, Russia: levels, temporal trends and infant exposure assessment. Chemosphere.

[CR114] Man YB, Chow KL, Xing GH, Chan JKY, Wu SC, Wong MH (2017). A pilot study on health risk assessment based on body loadings of PCBs of lactating mothers at Taizhou, China, the world’s major site for recycling transformers. Environ Pollut.

[CR115] Mandal SM, Bharti R, Porto WF, Gauri SS, Mandal M, Franco OL, Ghosh AK (2014). Identification of multifunctional peptides from human milk. Peptides.

[CR116] Marin S, Villalba P, Diaz-Ferrero J, Font G, Yusà V (2011). Congener profile, occurrence and estimated dietary intake of dioxins and dioxin-like PCBs in foods marketed in the Region of Valencia (Spain). Chemosphere.

[CR117] Martin RM, Gunnell D, Smith GD (2005). Breastfeeding in infancy and blood pressure in later life: systematic review and meta-analysis. Am J Epidemiol.

[CR118] Martί M, Ortiz X, Gasser M, Martί R, Montaña MJ, Dίaz-Ferrero J (2010). Persistent organic pollutants (PCDD/Fs, dioxin-like PCBs, marker PCBs, and PBDEs) in health supplements on the Spanish market. Chemosphere.

[CR119] Matos C, Ribeiro M, Guerra A (2015). Breastfeeding: antioxidative properties of breast milk. J Appl Biomed.

[CR120] Meki A-RMA, Saleem TA, Al-Ghazali MH, Sayed AA (2003). Interleukins -6, -8 and -10 and tumor necrosis factor-alpha and its soluble receptor I in human milk at different periods of lactation. Nutr Res.

[CR121] Mezynska M, Brzóska MM (2018). Environmental exposure to cadmium-a risk for health of the general population in industrialized countries and preventive strategies. Environ Sci Pollut Res Int.

[CR122] Mizuno K, Hatsuno M, Aikawa K, Takeichi H, Himi T, Kaneko A, Kodaira K, Takahashi H, Itabashi K (2012). Mastitis is associated with il-6 levels and milk fat globule size in breast milk. J Hum Lact.

[CR123] MohanKumar K, Namachivayam K, Ho TTB, Torres BA, Ohls RK, Maheshwari A (2017). Cytokines and growth factors in the developing intestine and during necrotizing enterocolitis. Semin Perinatol.

[CR124] Mokarizadeh A, Faryabi MR, Rezvanfar MA, Abdollahi M (2015). A comprehensive review of pesticides and the immune dysregulation: mechanisms, evidence and consequences. Toxicol Mech Methods.

[CR125] Morais TC, Honorio-França AC, Silva RR, Fujimori M, Fagundes DLG, França EL (2015). Temporal fluctuations of cytokine concentrations in human milk. Biol Rhythm Res.

[CR126] Műller MHB, Polder A, Brynildsrud OB, Lie E, Loken KB, Manyilizu WB, Mdegela RH, Mokiti F, Murtadha M, Nonga HE, Skaare JU, Lyche JL (2016). Brominated flame retardants (BFRs) in breast milk and associated health risks to nursing infants in Northern Tanzania. Environ Int.

[CR127] Needleman H (2004). Lead poisoning. Annu Rev Med.

[CR128] Nelson ED, McConnell LL, Baker JE (1998). Diffusive exchange of gaseous polycyclic aromatic hydrocarbons and polychlorinated biphenyls across the air-water Interface of the Chesapeake Bay. Environ Sci Technol.

[CR129] Nonqonierma AB, FlitzGerald RJ (2015). Bioactive properties of milk proteins in humans: a review. Peptides.

[CR130] Norstrӧm K, Czub G, McLachlan MS, Hu D, Thorne PS, Hornbuckle KC (2010). External exposure and bioaccumulation of PCBs in humans living in a contaminated urban environment. Environ Int.

[CR131] Nowak K, Ratajczak-Wrona W, Górska M, Jabłońska E (2018). Parabens and their effects on the endocrine system. Mol Cell Endocrinol.

[CR132] Ohno K, Yanase T, Matsuo Y, Kimura T, Rahman MH, Magara Y, Matsui Y (2007). Arsenic intake via water and food by a population living in an arsenic-affected area of Bangladesh. Sci Total Environ.

[CR133] Olivares M, Albrecht S, De Palma G, Ferrer MD, Castillejo G, Schols HA, Sanz Y (2015). Human milk composition differs in healthy mothers and mothers with celiac disease. Eur J Nutr.

[CR134] Owen CG, Whincup PH, Gilig JA, Cook DG (2003). Effect of breast feeding in infancy on blood pressure in later life: systematic review and meta-analysis. BMJ.

[CR135] Paliwoda RE, Newbigging AM, Wang Z, Le XC (2016). Benefits and risk associated with consumption of Great Lakes fish containing omega-3 fatty acids and polychlorinated biphenyls (PCBs). J Environ Sci (China).

[CR136] Pan S, Lin L, Zeng F, Zhang J, Dong G, Yang B, Jing Y, Schejun C, Zhang G, Zhiqiang Y, Sheng G, Ma H (2018). Effects of lead, cadmium, arsenic, and mercury co-exposure on children's intelligence quotient in an industrialized area of southern China. Environ Pollut.

[CR137] Park Y, Lee A, Choi K, Kim HJ, Lee JJ, Choi G, Kim S, Kim SY, Cho GJ, Suh E, Kim SK, Eun SH, Eom S, Kim S, Kim GH, Moon HB, Kim S, Choi S, Kim YD, Kim J, Park J (2018). Exposure to lead and mercury through breastfeeding during the first month of live: a CHECK cohort study. Sci Total Environ.

[CR138] Parvez S, Evans AM, Lorber M, Hawkins BS, Swartout JC, Teuschler LK, Rice GE (2013). A sensitivity analysis using alternative toxic equivalency factors to estimate U.S. dietary exposures to dioxin-like compounds. Regul Toxicol Pharmacol.

[CR139] Passatore L, Rossetti S, Juwarkar AA, Massacci A (2014). Phytoremediation and bioremediation of polychlorinated biphenyls (PCBs): state of knowledge and research perspectives. J Hazard Mater.

[CR140] Perellό G, Dίaz-Ferrero J, Llobet JM, Castell V, Vicente E, Nadal M, Domingo JL (2015). Human exposure to PCDD/Fs and PCBs through consumption of fish and seafood in Catalonia (Spain): temporal trend. Food Chem Toxicol.

[CR141] Polder A, Gabrielsen GW, Odland JØ, Savinova TN, Tkachev A, Løken KB, Skaare JU (2008). Spatial and temporal changes of chlorinated pesticides, PCBs, dioxins (PCDDs/PCDFs) and brominated flame retardants in human breast milk from Northern Russia. Sci Total Environ.

[CR142] Pratt IS, Anderson WA, Crowley D, Daly SF, Evans RI, Fernandes AR, Fitzgerald M, Geary MP, Keane DP, Malisch R, McBride J, Morrison JJ, Reilly A, Tlustos C (2012). Polychlorinated dibenzo-p-dioxins (PCDDs), polychlorinated dibenzofurans (PCDFs) and polychlorinated biphenyls (PCBs) in breast milk of first-time Irish mothers: impact of the 2008 dioxin incident in Ireland. Chemosphere.

[CR143] Prokesová L, Lodinová-Zádníková R, Zizka J, Kocourková I, Novotná O, Petrásková P, Sterzl I (2006). Cytokine levels in healthy and allergic mothers and their children during the first year of life. Pediatr Allergy Immunol.

[CR144] Quaqliato LA, Nardi AE (2017). Cytokine alteration in panic disorder: a systematic review. J Affect Disord.

[CR145] Radillo O, Norcio A, Addobbati R, Zauli G (2013). Presence of CTAK/CCL27, MCP-3/CCL7 and LIF in human colostrum and breast milk. Cytokine.

[CR146] Ragib R, Ahmed S, Sultana R, Wagatsuma Y, Mondal D, Hoque AM, Nermell B, Yunus M, Roy S, Persson LA, Arifeen SE, Moore S, Vahter M (2009). Effects of in utero arsenic exposure on child immunity and morbidity in rural Bangladesh. Toxicol Lett.

[CR147] Rawn DF, Breakell K, Vergin V, Nicolidakis H, Sit D, Feeley M (2009). Persistent organic pollutants in fish oil supplements on the Canadian market: polychlorinated biphenyls and organochlorine insecticides. J Food Sci.

[CR148] Rebelo FM, Caldas ED (2016). Arsenic, lead, mercury and cadmium: toxicity, levels in breast milk and the risks for breastfed infants. Environ Res.

[CR149] Rebelo FM, Cunhna LRD, Andrade PD, Costa Junior WAD, Bastos WR, Caldas ED (2017). Mercury in breast milk from women in the Federal District, Brazil and dietary risk assessment for breastfed infants. J Trace Elem Med Biol.

[CR150] Rentea RM, Wagner AJ, Gourlay DM, Christensen M, Liedel JL (2017). Effects of anticipated neonatal surgical intervention on maternal milk cytokine production. J Pediatr Surg.

[CR151] Ribas-Fitό N, Torrent M, Carrizo D, Munoz-Ortiz L, Julvez J, Grimalt JO, Sunyer J (2006). In utero exposure to background concentrations of DDT and cognitive functioning among preschoolers. Am J Epidemol.

[CR152] Rigotti E, Piacentini GL, Ress M, Pigozzi R, Boner AL, Peroni DG (2006). Transforming growth-factor-beta and interleukin-10 in breast milk and development of atopic diseases in infants. Clin Exp Allergy.

[CR153] Ritter R, Scheringer M, MacLeod M, Moeckel M, Jones KC, Hungerbühler K (2011). Intrinsic human elimination half-lives of polychlorinated biphenyls derived from the temporal evolutio3n of cross-sectional biomonitoring data from the United Kingdom. Environ Health Perspect.

[CR154] Rivezzi G, Piscitelli P, Scortichini G, Giovannini A, Diletti G, Migliorati G, Ceci R, Rivezzi G, Cirasino L, Carideo P, Black DM, Garzillo C, Giani U (2013). A general model of dioxin contamination in breast milk: results from a study on 94 women from the Caserta and Naples areas in Italy. Int J Environ Res Public Health.

[CR155] Rocheleau CM, Bertke SJ, Deddens JA, Ruder AM, Lawson CC, Waters MA, Hopf NB, Riggs MA, Whelan E (2011). Maternal exposure to polychlorinated biphenyls and the secondary sex ratio: an occupational cohort study. Environ Health.

[CR156] Rochester JR (2013). Bisphenol A and human health: a review of the literature. Reprod Toxicol.

[CR157] Rowley B, Monestier M (2005). Mechanisms of heavy metal-induced autoimmunity. Mol Immunol.

[CR158] Ryan JJ, Rawn DF (2014). Polychlorinated dioxins, furans (PCDD/Fs), and polychlorinated biphenyls (PCBs) and their trends in Canadian human milk from 1992 to 2005. Chemosphere.

[CR159] Sakamoto M, Chan HM, Domingo JL, Kubota M, Murata K (2012). Changes in body burden of mercury, lead, arsenic, cadmium and selenium in infants during early lactation in comparison with placental transfer. Ecotoxicol Environ Saf.

[CR160] Samiee F, Vahidinia A, Javad MT, Leili M (2019). Exposure to heavy metals released to the environment through breastfeeding. A probabilistic risk estimation. Sci Total Environ.

[CR161] Sarkar A, Ravindran G, Krishnamurthy V (2013). A brief review on the effect of cadmium toxicity: from cellular to organ level. Int J Adv Biotechnol Res.

[CR162] Sattar A, Xie S, Hafeez MA, Wang X, Hussain HI, Iqbal Z, Pan Y, Iqbal M, Shabbir MA, Yuan Z (2016). Metabolism and toxicity of arsenicals in mammals. Environ Toxicol Pharmacol.

[CR163] Schuhmacher M, Kiviranta H, Vartiainen T, Domingo JL (2007). Concentrations of polychlorinated biphenyls (PCBs) and polybrominated diphenyl ethers (PBDEs) in milk of women form Catalonia, Spain. Chemosphere.

[CR164] Shaban NS, Abdou KA, Hassan NEHY (2016). Impact of toxic heavy metals and pesticide residues in herbal products. Beni-Suef Univ. J Appl Sci.

[CR165] Shelton JF, Geraghty EM, Tancredi DJ, Delwiche LD, Schmidt RJ, Ritz B, Hansen RL, Hertz-Picciotto I (2014). Neurodevelopmental disorders and prenatal residential proximity to agricultural pesticides: the CHARGE study. Environ Health Perspect.

[CR166] Sinicropi MS, Amantea D, Caruso A, Saturnino C (2010). Chemical and biological properties of toxic metals and use of chelating agents for the pharmacological treatment of metal poisoning. Arch Toxicol.

[CR167] Skrbić B, Szyrwińska K, Durišić-Mladenović N, Nowicki P, Lulek J (2010). Principal component analysis of indicator PCBs profiles in breast milk from Poland. Environ Int.

[CR168] Sowers MR, Scholl TO, Hall G, Jannausch ML, Kemp FW, Li X, Bogden JD (2002). Lead in breast milk and maternal bone turnover. Am J Obstet Gynecol.

[CR169] Stenken JA, Poschenrieder AJ (2015). Bioanalytical chemistry of cytokines-a review. Anal Chim Acta.

[CR170] Sternowsky HJ, Moser B, Szadkowsky D (2002). Arsenic in breast milk during the first 3 months of lactation. Int J Hyg Environ Health.

[CR171] Struciński P, Piskorska-Pliszczyńska J, Maszewski S, Góralczyk K, Warenik-Bany M, Mikołajczyk SZ, Czaja K, Hernik A, Ludwicki JK (2013). PCDD.Fs and DL-PCBs intake from fish caught in Polish fishing grounds in the Baltic Sea - characterizing the risk for consumers. Environ Int.

[CR172] Szyrwińska K, Lulek J (2007). Exposure to specific polychlorinated biphenyls and some chlorinated pesticides via breast milk in Poland. Chemosphere.

[CR173] Tan CC, Yu JT, Tan L (2014). Biomarkers for preclinical Alzheimer's disease. J Alzheimers Dis.

[CR174] Tang-Péronard JL, Heitmann BL, Andersen HR, Steuerwald U, Grandjean P, Weihe P, Jensen TK (2014). Association between prenatal polychlorinated biphenyl exposure and obesity development at ages 5 and 7 y: a prospective cohort study of 656 children from the Faroe Islands. Am J Clin Nutr.

[CR175] Taylor KW, Novak RF, Anderson HA, Birnbaum LS, Blystone C, De Vito M, Jacobs D, Kőgrle J, Lee D-H, Rylander L, Rignell-Hydrbom A, Tornero-Velez R, Turyk ME, Boyles AL, Thayer KA, Lind LE (2013). Evaluation of the association between persistent organic pollutants (POPs) and diabetes in epidemiological studies: a national toxicology program workshop review. Environ Health Perspect.

[CR176] Taylor V, Goodale B, Raab A, Schwerdtle T, Reimer K, Conklin S, Karagas MR, Francesconi KA (2017). Human exposure to organic arsenic species from seafood. Sci Total Environ.

[CR177] Thomas Curtis J, Chen Y, Buck DJ, Davis RL (2011). Chronic inorganic mercury exposure induces sex-specific changes in central TNFα expression: importance in autism?. Neurosci Lett.

[CR178] Tirisina A, Sircu R, Pinzaru I, Bahnarel I (2017). Changes over time in persistent pollutants (POP) concentrations in human milk in the Republic of Moldova. Toxicol Environ Chem.

[CR179] Tripathi RM, Raqhunath R, Sastry VN, Krishnamoorthy TM (1999). Daily intake of heavy metals by infants through milk and milk products. Sci Total Environ.

[CR180] Tuaillon E, Viljoen J, Dujols P, Cambonie G, Rubbo PA, Nagot N, Bland RM, Badiou S, Newell ML, Van de Perre P (2017). Subclinical mastitis occurs frequently in association with dramatic changes in inflammatory/anti-inflammatory breast milk components. Pediatr Res.

[CR181] Uemura H, Arisawa K, Hiyoshi M, Satoh H, Sumiyoshi Y, Morinaga K, Kodama K, Suzuki T, Nagai M, Suzuki T (2008). PCDDs/PCDFs and dioxin-like PCBs: Recent body burden levels and their determinants among general inhabitants in Japan. Chemosphere.

[CR182] Urbaniak M, Kiedrzyńska E, Kiedrzyński M, Zieliński M, Grochowalski A (2015). The role of hydrology in the polychlorinated dibenzo-p-dioxin and dibenzofuran distributions in a lowland river. J Environ Qual.

[CR183] Vahter M (2002). Mechanisms of arsenic biotransformation. Toxicology.

[CR184] Van den Berg M, Birnbaum LS, Denison M, De Vito M, Farland W, Feeley M, Feeley M, Hakansson H, Hanberg A, Haws L, Rose M, Safe S, Schrenk D, Tohyama C, Tritscher A, Tuomisto J, Walker N, Peterson RE (2006). The 2005 World Health Organization reevaluation of human and mammalian toxic equivalency factors for dioxins and dioxin-like compounds. Toxicol Sci.

[CR185] Van den Berg M, Denison MS, Birnbuam LS, DeVito MJ, Fiedler H, Falandysz J, Rose M, Schrenk D, Safe S, Tohyama C, Tritscher A, Tysklind M, Peterson RE (2013). Polybrominated dibenzo-pdioxins, dibenzofurans, and biphenyls: inclusion in the toxicity equivalency factor concept for dioxin-like compounds. Toxicol Sci.

[CR186] Van den Berg M, Kypke K, Kotz A, Tritscher A, Lee SY, Magulova K, Fiedler H, Malisch R (2017). WHO/UNEP global surveys of PCDDs, PCDFs, PCBs and DDTs in human milk and benefit-risk evaluation of breastfeeding. Arch Toxicol.

[CR187] Vennemann MM, Loddenkӧtter B, Fracasso T, Mitchell EA, Debertin AS, Larsch KP, Sperhake JP, Brinkmann B, Sauerland C, Lindemann M, Bajanowski T (2012). Cytokines and sudden infant death. Int J Legal Med.

[CR188] Verner M-A, Plusquellec P, Desjardins JL, Cartier C, Haddad S, Ayotte P, Dewaily E, Muckle G (2015). Prenatal and early-life polychlorinated biphenyl (PCB) levels and behavior in Inuit preschoolers. Environ Int.

[CR189] Vigh É, Colombo A, Benfenati E, Håkansson H, Berglund M, Bódis J, Garai J (2013). Individual breast milk consumption and exposure to PCBs and PCDD/Fs in Hungarian infants: a time-course analysis of the first three months of lactation. Sci Total Environ.

[CR190] Waalkes MP (2000). Cadmium carcinogenesis in review. I Inorg Biochem.

[CR191] Walker A (2010). Breast milk as the gold standard for protective nutrients. J Pediatr.

[CR192] Wang DC, Yu P, Zhang Y, Cui Y, Sun CH (2008). The determination of persistent organic pollutants (POPs) in the colostrums of women in preterm labor. Clin Chim Acta.

[CR193] Wang H, Liu L, Hu YF, Hao JH, Chen YH, Su PY, Yu Z, Fu L, Tao FB, Xu DX (2016). Association of maternal serum cadmium level during pregnancy with risk of preterm birth in a Chinese population. Environ Pollut.

[CR194] Wani AL, Ara A, Usmani JA (2015). Lead toxicity: a review. Interdiscip Toxicol.

[CR195] WHO (2010). Childhood Lead Poisoning.

[CR196] Winiarska-Mleczan A (2014). Cadmium, lead, copper and zinc in breast milk in Poland. Biol Trace Elem Res.

[CR197] Winkler B, Aulenbach J, Meyer T, Wiegering A, Eyrich M, Schlegel PG, Wiegering V (2015). Formula-feeding is associated with shift towards Th1 cytokines. Eur J Nutr.

[CR198] Wong M-H (2017). Chemical pollution and seafood safety, with a focus on mercury: the case of Pearl River Delta, South China. Environ Technol Innov.

[CR199] Yamazaki K, Araki A, Nakajima S, Miyashita C, Ikeno T, Itoh S, Minatoya M, Kobayashi S, Mizutani F, Chisaki Y, Kishi R (2018). Association between prenatal exposure to organochlorine pesticides and the mental and psychomotor development of infants at ages 6 and 18 months. The Hokkaido Study on Environment and Children's Health. Neurotoxicology.

[CR200] Zalups RK, Ahmed S (2003). Molecular handling of cadmium in transporting epithelia. Toxicol Appl Pharmacol.

[CR201] Zanardo V, Nicolssi S, Cavallin S, Trevisanuto D, Barbato A, Faggian D, Favaro F, Plebani M (2005). Effect of maternal smoking on breast milk interleukin-1α, β-endorphin, and leptin concentrations. Environ Health Perspect.

[CR202] Zanardo V, Golin R, Armato M, Trevisanuto D, Favaro F, Faggian D, Plebani M (2007). Cytokines in human colostrum and neonatal jaundice. Pediatr Res.

[CR203] Zhang L, Yin S, Li J, Zhao Y, Wu Y (2016). Increase of polychlorinated dibenzo-p-dioxins and dibenzofurans and dioxin-like polychlorinated biphenyls in human milk from China in 2007-2011. Int J Hyg Environ Health.

[CR204] Zhang L, Shuaixing Y, Zhao Y, Shi Z, Li J, Wu Y (2017). Polybrominated diphenyl ethers and indicator polychlorinated biphenyls in human milk from China under the Stockholm Convention. Chemosphere.

[CR205] Zizka J, Kverka M, Novotná O, Stanková I, Lodinová-Zádníková R, Kocourková I, Sterzl I, Prokesová L (2007). Perinatal period cytokines related to increased risk of future allergy development. Folia Microbiol (Praha).

